# Drp1 and RB interaction to mediate mitochondria-dependent necroptosis induced by cadmium in hepatocytes

**DOI:** 10.1038/s41419-019-1730-y

**Published:** 2019-07-08

**Authors:** Shili Zhang, Lin Che, Chengyong He, Jing Huang, Nijun Guo, Jiazhang Shi, Yuchun Lin, Zhongning Lin

**Affiliations:** State Key Laboratory of Molecular Vaccinology and Molecular Diagnostics, School of Public Health, Xiamen University, Xiamen, 361102 China

**Keywords:** Necroptosis, Necroptosis, Mechanisms of disease, Mechanisms of disease

## Abstract

Mitochondrial quality control (MQC) is implicated in cell death induced by heavy metal pollutants. Dynamin-related protein 1 (Drp1) regulates mitochondrial fission, which is an important part of MQC. Retinoblastoma (RB) protein can regulate MQC in a transcription-independent manner. Necroptosis plays a critical role in hepatic pathologies such as inflammatory, infectious, and xenobiotics-induced injury and diseases. We aimed to explore the role and mechanism of Drp1 interaction with RB in hepatocyte’s necroptosis caused by cadmium (Cd). CdCl_2_ was employed to expose to Institute of Cancer Research (ICR) mice and human hepatic L02 cells. CdCl_2_ exposure induced necroptosis and hepatic injury both in vivo and in vitro. Moreover, Drp1 and RB protein were up-regulated and translocated to mitochondria in CdCl_2_-exposed hepatocytes. Inhibition of Drp1 with siRNA (si*DNM1L*) or inhibitors not only suppressed the RB expression and its mitochondrial translocation, but also alleviated MQC disorder, necroptosis, and hepatotoxicity caused by CdCl_2_. Moreover, blocking Drp1 with metformin rescued necroptosis and hepatic injury triggered by CdCl_2_. RB was proved to directly interact with Drp1 at mitochondria to form a complex which then bound to receptor interaction protein kinase (RIPK3) and enhanced the formation of necrosome after CdCl_2_ exposure. In summary, we found a new molecular mechanism of regulated cell death that Drp1 interacted with RB and promoted them mitochondrial translocation to mediate necroptosis and hepatic injury in hepatocytes induced by Cd-exposure. The mitochondrial Drp1-RB axis would be a novel target for the protection cells from xenobiotics triggering hepatic injury and diseases involved in necroptosis.

## Introduction

Necroptosis, one type of regulated cell deaths, can be stimulated by ligands that bind to tumor necrosis factor (TNF) family death domain receptors, pattern recognizing receptors, and virus sensors^1,2^. These receptor systems often trigger downstream key proteins, including receptor interaction protein kinase (RIPK) 1, RIPK3, and mixed lineage kinase domain like (MLKL) protein^3,4^. Specifically, once RIPK1 recruits and activates RIPK3 through RIPK homotypic interaction motif, RIPK3 will phosphorylate MLKL (p-MLKL) at the threonine 357 (Thr357) and serine 358 (Ser358) sites^3^, leading to exposure of the N-terminal four helical bundle domain, which finally binds to phosphatidylinositol lipids and cardiolipin^5^. This feature allows the transfer of MLKL from cytosol to cell plasma membrane and organelles’ membranes, where it directly disrupts membrane integrity, releases damage-associated molecular patterns, and results in necroptosis^6^. Besides the phosphorylation regulatory mechanism, we aimed to explore other molecular events that might regulate the execution of necroptosis.

Recent studies have shown that necroptosis plays a critical role in human pathologies including many inflammatory, degenerative, and infectious diseases, especially in xenobiotics-induced liver injury and diseases^2,7,8^. The expression of RIPK3 is higher in the liver of patients with alcoholic liver disease than that of normal people^9^. Moreover, RIPK3-deficient mice present less hepatitis, liver injury, and steatosis after alcohol administration^9^. On the other hand, necroptosis-related diseases can be triggered by xenobiotics exposure, such as cigarette smoking, nanomaterials, and environmental heavy metal pollutants^8,10,11^. For example, cadmium (Cd) increases the mRNA levels of *RIPK1* and *MLKL* in chicken cardiomyocytes^12^, and induces necroptosis in rainbow trout cells derived from gill^13^. Cd and its compounds (Cd^2+^) are common heavy metal contaminant, mainly from industrial productions, such as mining, alloy manufacturing, electroplating, and rechargeable batteries. Human Cd exposure mainly comes from environment^14^. Diet and lifestyle are primarily exposure pathways, such as drinking Cd-containing water and inhalation of cigarette smoking^15^. Liver is one of the main targeted organs for acute or chronic Cd exposure. Many studies reported that Cd could induce autophagy, apoptosis, and necrosis in hepatocytes^16–18^. However, it is still obscure whether Cd can induce necroptosis in hepatocytes or not and its mechanisms involved in mitochondrial quality control (MQC) remain to be clarified.

MQC is extremely important for maintaining mitochondrial homeostasis through autophagy, mitochondria-related proteins distribution, and mitochondrial dynamics^19,20^. Mitochondrial dynamics, including mitochondrial fusion and fission, is regulated by dynamin-related protein 1 (Drp1), mitofusin (Mfn) 1 and Mfn2, and other factors^21^. Once damage is irreversible, mitochondria will present excessive fission, fragmentation, mass decline, and membrane integrity loss^22^. Drp1, encoded by *DNM1L* gene, is a cytosolic protein that can be recruited to the mitochondrial outer membrane (MOM) by interacting with mitochondrial receptor proteins (e.g., mitochondrial fission factor and fission protein 1) to mediate mitochondrial fission^23,24^. Interestingly, many studies have reported that the necroptosis-related proteins are located at mitochondria and interact with Drp1^25,26^. Wang found that mitochondrial damage preceded necroptosis occurrence and RIPK1/RIPK3 complex directly phosphorylated Drp1 at serine 616 site (p-Drp1^(Ser616)^) and triggered its translocation to mitochondria^2^. P-MLKL also could be translocated to mitochondrial membrane when necroptosis occurred in human colon cancer HT-29 cells^25^. These studies suggest that MQC, regulated by Drp1, might be an intermediate event of necroptosis.

Retinoblastoma (RB) protein, encoded by *RB1* gene, is a transcriptional co-regulator in many cellular processes^27^. Its mechanisms are mainly divided into transcription-dependent and -independent manners. The nuclear RB generally binds to E2F transcription factor 1 to block cell cycle, leading to decrease of proliferation^27^. On the other hand, RB is also located at cytoplasm and can be translocated to mitochondria to exert some transcription–independent functions^28^. For example, RB promotes mitochondria-dependent apoptosis and oxidative phosphorylation, which can be blocked by RB mutation at serine 807/811 sites^29,30^. RB also regulates the mitochondrial protein expression and redox status, both of which are important for mitochondrial homeostasis^31,32^. In current study, human immortalized hepatic L02 cell line and Institute of Cancer Research (ICR) mice (one of the commonly available outbred population because they have good reproductive performance, are inexpensive, robust, and grow rapidly, and have been widely used in various research fields including toxicology^33^ were treated by CdCl_2_ both in vitro and in vivo, respectively. We aimed to investigate whether RB participated in Drp1-mediated MQC alteration to regulate necroptosis and hepatic injury caused by xenobiotics exposure.

## Results

### CdCl_2_ induces injury and necroptosis of hepatocytes both in vivo and in vitro

**Fig. 1:**
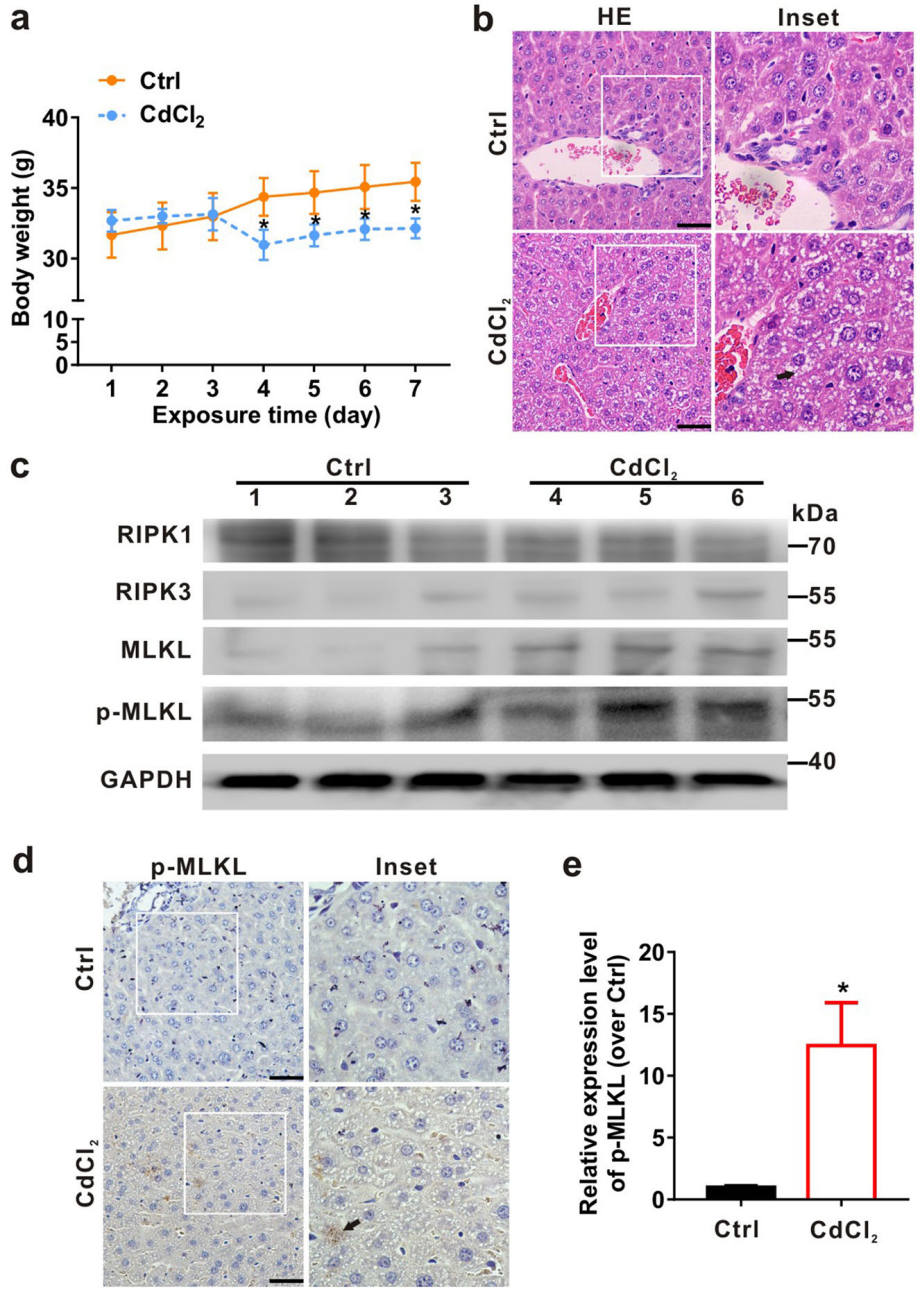
CdCl_2_ induces necroptosis in liver tissues of ICR mice. The ICR mice were intraperitoneally injected with CdCl_2_ (1 mg/kg, *n* = 3) or physiological saline (Ctrl group, *n* = 3) every day for one week. **a** Body weight curve. **b** Histological changes (HE staining) of liver. Arrow indicates the histological changes (hepatic cord incomplete and vacuolation) of the liver. **c** The levels of necroptosis-related proteins (RIPK1, RIPK3, MLKL, and p-MLKL) were analyzed by Western blot. **d**, **e** Liver tissues were subjected to immunohistochemistry (IHC) using p-MLKL antibody. **d** The representative images of IHC were shown. Arrow indicates the p-MLKL signals in the liver. The scale bar is 50 μm. **e** The images were semi-quantified using ImagePro-plus 6.0. Five fields of view were calculated for each group. Data are expressed as the mean ± standard deviation (SD). *P* < 0.05, *significantly different from Ctrl

To investigate the role of necroptosis in hepatotoxicity, we established an acute CdCl_2_ exposure model based on our previous study^33^. Adult ICR mice were selected and intragastrically administered with physiological saline (as control, Ctrl group) or 1 mg/kg CdCl_2_ every day for one week. The body weight of mice in the CdCl_2_ group had significant reduction from the fourth day compared to Ctrl group (Fig. 1a). As shown in Fig. 1b, incomplete hepatic cord and vacuolation of mice liver were observed in the CdCl_2_ group using HE staining. Furthermore, necroptosis-related proteins were detected using Western blot and IHC analyses. Compared to Ctrl group, the protein levels of RIPK3 and p-MLKL were increased, while the RIPK1 was decreased in the CdCl_2_ group (Fig. 1c). The semi-quantitative analysis for IHC showed that the brown density of p-MLKL was increased about 11.43-fold in the CdCl_2_ group compared to that in Ctrl group (Fig. 1d, e). These results indicated that CdCl_2_ induced hepatic injury and necroptosis in vivo.

**Fig. 2:**
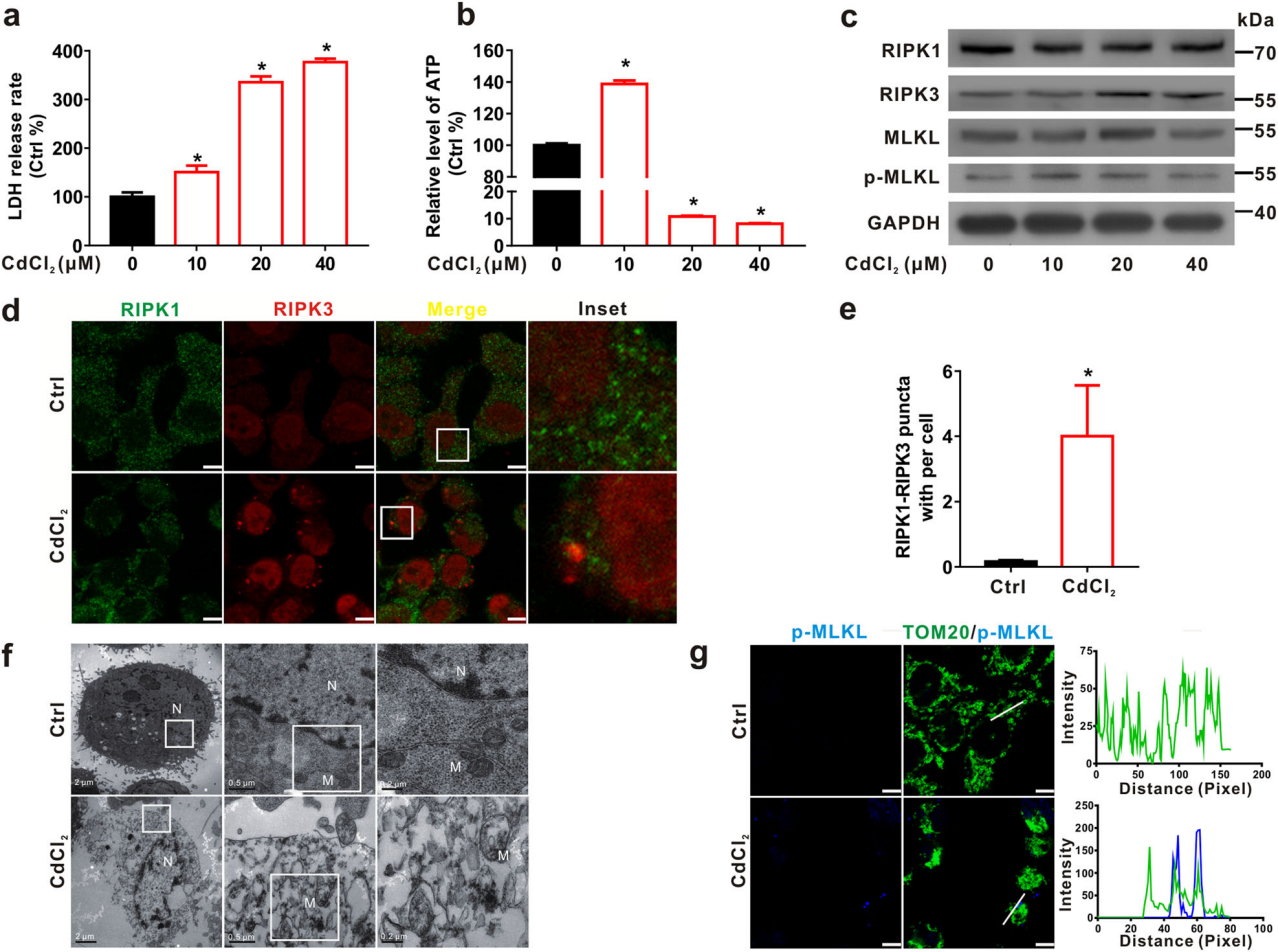
CdCl_2_ induces necroptosis in L02 cells. **a**, **b** L02 cells were exposed to different concentrations of CdCl_2_ for 6 h. **a** Membrane integrity was detected by LDH release assay. **b** ATP concentration was detected using an ATP Assay Kit. **c** Necroptosis-related proteins were evaluated by Western blot after L02 cells were exposed to CdCl_2_ with different concentrations as indicated. **d**, **e** L02 cells were treated with CdCl_2_ (20 μM) for 6 h. Cells were subjected to IF staining with RIPK1 antibody (green) and RIPK3 antibody (red). **d** The representative IF images were shown. The scale bar is 10 μm. **e** Quantification of RIPK1-RIPK3 puncta per cell was conducted using ImagePro-plus 6.0. **f** Ultrastructure of cells was observed by TEM. The scale bars are 2, 0.5, and 0.2 μm, respectively. *M* mitochondrion; *N* nucleus. **g** Cells were subjected to IF staining with p-MLKL antibody (blue) and TOM20 antibody (green). The profiles of representative lines trace the intensities of p-MLKL signals along with TOM20. Fluorescence curves with line intensity profile generated by Zen 2012 software were shown. The scale bar is 10 μm. Data are expressed as the mean ± standard deviation (SD). *P* < 0.05, *significantly different from Ctrl

In vitro, human immortalized hepatic L02 cells were exposed to CdCl_2_ at different concentrations (10, 20, and 40 μM) for 6 h or at 20 μM for various durations (3, 6, and 12 h). At the beginning, we tested whether L02 cells went through necroptosis or not. TNF-α plus pancaspase inhibitor z-VAD is a well-known inducer of necroptosis^34^. In Supplementary Fig. S1a, we found that necroptosis could be induced by TNF-α plus z-VAD in L02 cells. CdCl_2_ induced p-MLKL up-regulation was inhibited by MLKL protein inhibitor necrosulfonamide (NSA) plus z-VAD (Supplementary Fig. S1b). The cell viability had no significant change when cells were treated with CdCl_2_ plus either z-VAD or NSA compared to CdCl_2_ group. The necroptosis induced by CdCl_2_ was inhibited only by NSA plus z-VAD (Supplementary Fig. S1c). When MLKL translocates to cell membrane, the intracellular osmotic pressure will increase, and ultimately leading to membrane rupture^35^. As expected, the release rate of lactate dehydrogenase (LDH) (Fig. 2a), and the levels of RIPK3 and p-MLKL proteins (Fig. 2c) were elevated and the content of ATP was reduced (Fig. 2b) by CdCl_2_ in a concentration-dependent manner. Consistently, CdCl_2_ exposure resulted in the decrease of ATP and the increase of RIPK3 and p-MLKL proteins in a time-dependent manner (Supplementary Fig. S2a, b). RIPK1, however, presented a downtrend which was in line with the result (Fig. S2b). The reason of RIPK1 were decreased by CdCl_2_ might be that reactive oxygen species (ROS) caused RIPK1 oxidation to form intermolecular disulfide bonds, resulting in that RIPK1 could not be detected in β-mercaptoethanol-denatured proteins^36^. Immunofluorescence (IF) assay showed that the fluorescence puncta of RIPK1 and RIPK3 were up-regulated by CdCl_2_ exposure (Fig. 2d, e). Moreover, we observed the typical features of necroptosis, including mitochondrial swelling, rupture of membranes, and cellular content release, by transmission electron microscope (TEM) in the CdCl_2_ group (Fig. 2f). To further explore the relationship of mitochondria with necroptosis caused by CdCl_2_, the co-localization of p-MLKL with mitochondrial membrane, IF staining of p-MLKL and TOM20 (a marker for MOM) was conducted. As shown in Fig. 2g, p-MLKL signals partially overlapped with MOM. In general, these results indicated that CdCl_2_ exposure induced mitochondria-dependent necroptosis of hepatocytes both in vivo and in vitro.

### Mitochondrial translocation of necrosome is accompanied by MQC disorder triggered by CdCl_2_

**Fig. 3:**
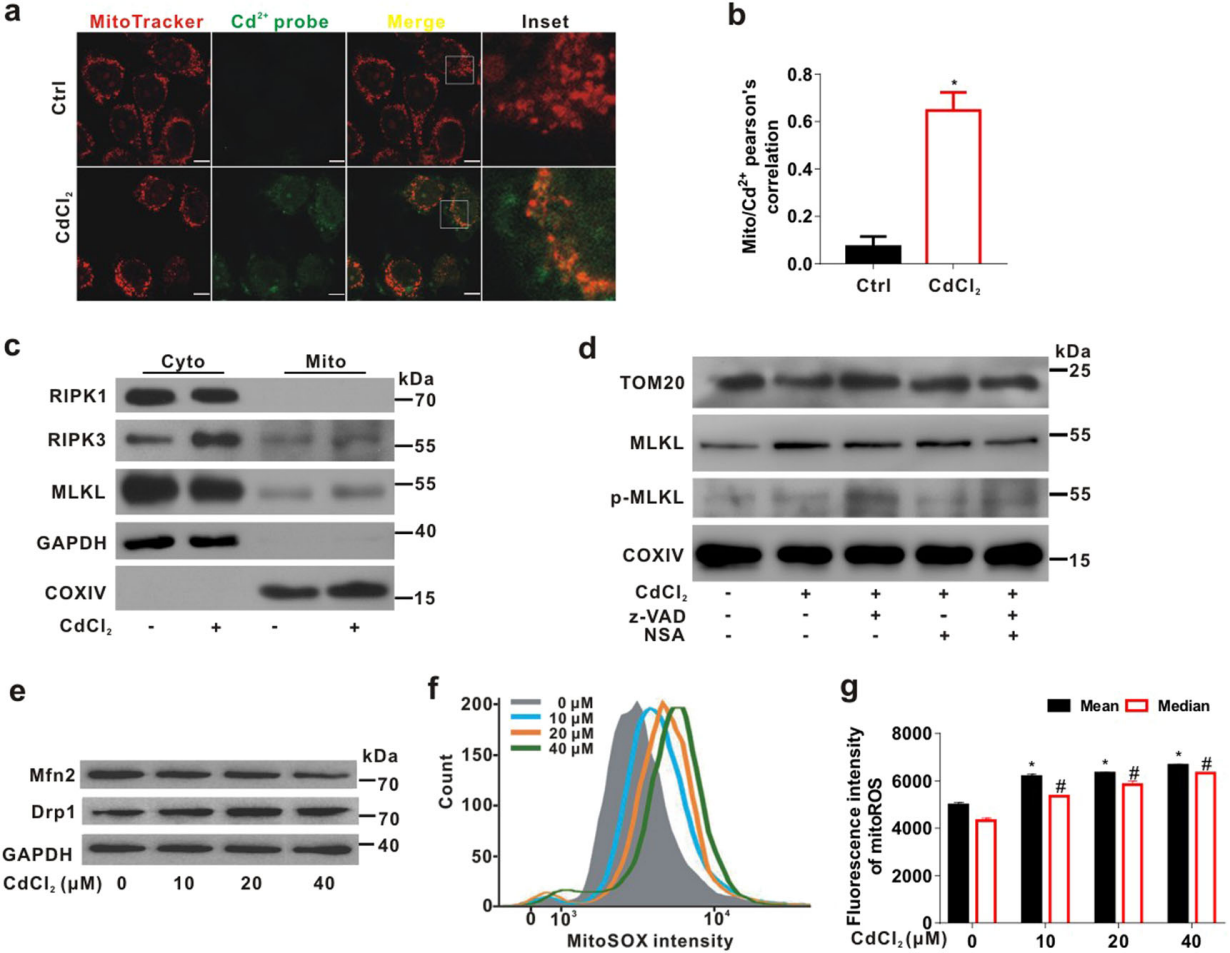
Mitochondria-distributed Cd^2+^ induces necrosome translocation to mitochondria and MQC disorder in L02 cells. **a**, **b** L02 cells were stained with MitoTracker Deep Red (red) and Leadmium™ Green AM Dye (Cd^2+^ probe; green) for mitochondria and intracellular Cd^2+^ and imaged using a confocal microscope. **a** The representative IF images were shown. The scale bar is 10 μm. **b** Mito/Cd^2+^ signal overlap of colocalization was analyzed with Pearson’s correlation using ImagePro-plus 6.0. Ten fields of view were calculated for each group. *P* < 0.05, *significantly different from Ctrl. **c** Cytoplasmic (Cyto) and mitochondrial (Mito) fractions were immunoblotted with the indicated antibodies. GAPDH or COXIV was used as loading control of cytoplasmic or mitochondrial fraction, respectively. **d** L02 cells were treated with CdCl_2_ (20 μM) and/or z-VAD (20 μM) and/or NSA (1 μM) for 6 h. Mitochondrial fractions were immunoblotted with the indicated antibodies. **e** L02 cells were exposed to CdCl_2_ (10, 20, and 40 μM) for 6 h. Mitochondrial dynamics-related proteins were immunoblotted with the indicated antibodies. **f**, **g** The mitoROS of L02 cells was detected by flow cytometry analysis after CdCl_2_ treatment for 6 h. **f** The flow cytometry images of mitoROS were shown. **g** Quantification of mitoROS fluorescence intensity was shown in bar charts. Data are expressed as the mean ± SD. *n* = 4. *P* < 0.05, *,^#^significantly different from the mean and median of Ctrl, respectively

MQC is one of important indexes to reflect the integrity of mitochondrial function^21^. We further investigated whether CdCl_2_ exposure directly targeted mitochondria using MitoTracker (red) and Cd^2+^ probe (green) co-staining. The colocalization coefficient was about 0.65 (*P* < 0.05) between mitochondria and Cd^2+^ after CdCl_2_ exposure (Fig. 3a, b). Mitochondrial cytochrome c oxidase IV (COXIV), one of key proteins encoded by mitochondrial DNA, is an important component of the mitochondrial respiratory chain. The level of COXIV indicates mitochondrial mass and can be used as loading control of mitochondrial protein^17^. The up-regulation of RIPK3 and MLKL was observed in the mitochondrial fraction from CdCl_2_-exposed cells (Fig. 3c). We also found that inhibition of MLKL with NSA suppressed CdCl_2_-induced MLKL and p-MLKL translocation to mitochondria under z-VAD treatment (Fig. 3d).

On the other hand, CdCl_2_ reduced the ratio of elongated mitochondria in L02 cells (Supplementary Fig. S3a, b). Moreover, Western blot analysis revealed that CdCl_2_ exposure led to the decrease of Mfn2 and the increase of Drp1 in a concentration-dependent manner (Fig. 3e). These results indicated the increase of mitochondrial fission and the disorder of mitochondrial dynamics triggered by CdCl_2_. In addition, mitochondrial reactive oxygen species (mitoROS) was significantly increased by CdCl_2_ exposure using flow cytometry (Fig. 3f, g). We further measured mitochondrial membrane potential (Δψm) and mitochondrial Ca^2+^ in CdCl_2_-treated L02 cells. As shown in Supplementary Fig. S3c, e, Δψm was dropped in concentration- and time-dependent manner. Also, mitochondrial Ca^2+^ was increased by CdCl_2_ exposure (Supplementary Fig. S3d, f). These data suggested that CdCl_2_ induced necrosome translocation to mitochondria and led to MQC disorder related to the mitochondrial fission in hepatocytes.

### RB enhances the formation of necrosome induced by CdCl_2_

**Fig. 4:**
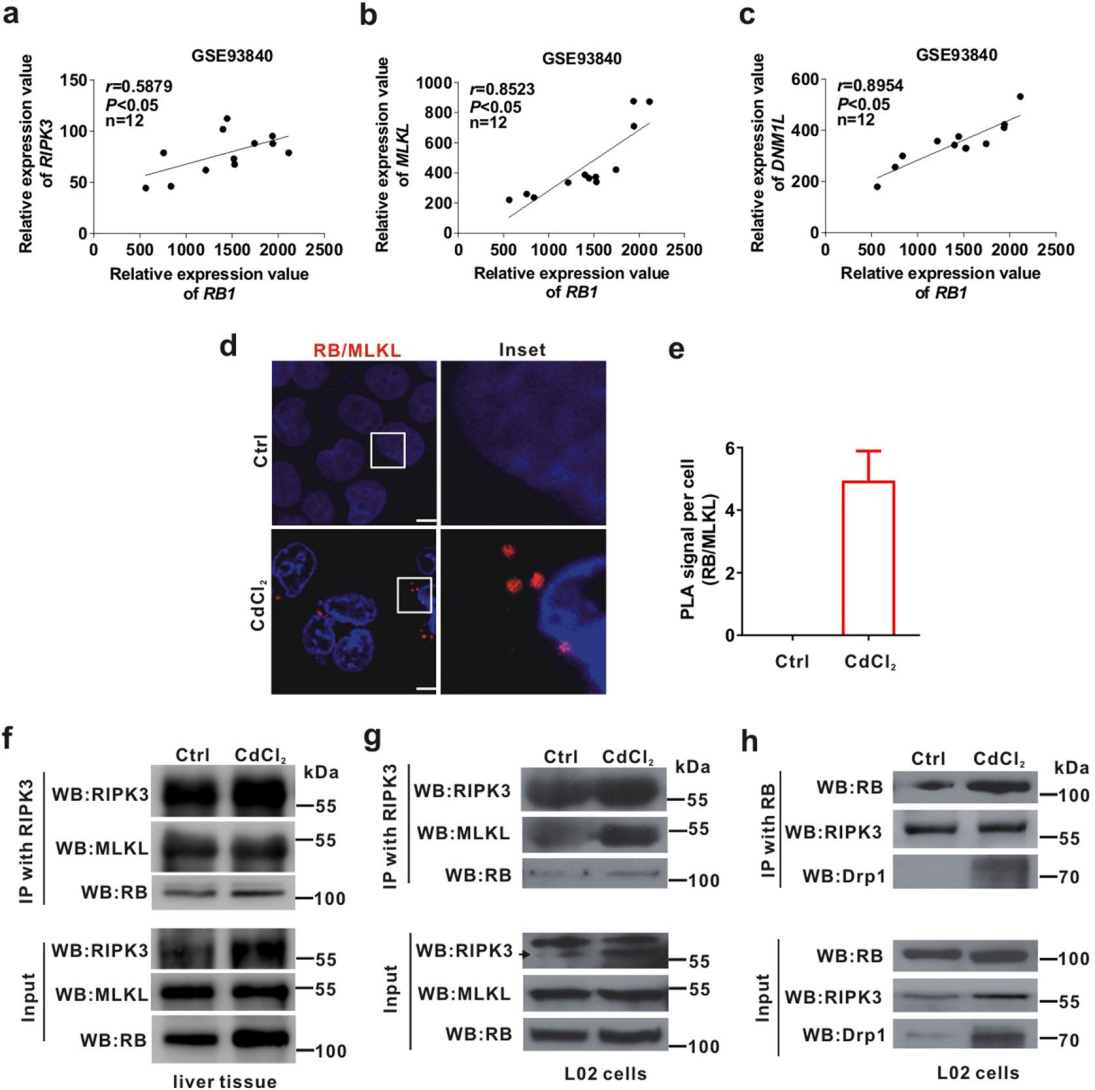
RB binds to Drp1 and promotes the formation of necrosome. **a**-**c** The linear correlation of the expression of *RB1* with *RIPK3*, *MLKL*, and *DNM1L* expression in primary human hepatocytes exposed to three xenobiotics (aflatoxin B1, amiodarone, and chlorpromazine) for 14 days from NCBI, GEO database (Accession No. GSE93840) was calculated. **d, e** PLA was used for detecting interaction between RB and MLKL. Nuclei were stained with 4',6-diamidino-2-phenylindole (DAPI). The scale bar is 10 μm. **d** Typical red foci of PLA indicated the protein-protein interaction between RB and MLKL. **e** Quantification of PLA foci per cell was shown in bar charts quantified using ImagePro-plus 6.0. Ten fields of view were calculated for each group. **f** The ICR mice (*n* = 3) were intraperitoneally injected with CdCl_2_ (1 mg/kg bw) or physiological saline (Ctrl group, *n* = 3) for 7 days. The mitochondrial fraction of liver was immunoprecipitated with anti-RIPK3 antibody, and then both IP and whole cell lysates (Input) were immunoblotted with the indicated antibodies. **g**, **h** L02 cells were treated with CdCl_2_ (20 μM) for 6 h. The whole cell lysates were immunoprecipitated with anti-RIPK3 or anti-RB antibody, and then both IP and whole cell lysates (Input) were immunoblotted with the indicated antibodies. Data are expressed as the mean ± standard deviation (SD). *P* < 0.05, *significantly different from Ctrl

It is clear that Drp1 and RIPK3 are required for TNF-α-induced necroptosis^4^. To elucidate whether RB interacts with Drp1 or RIPK3 to participate in the formation of necrosome, Gene Expression Omnibus (GEO) database analysis and Co-IP assay were conducted. We analyzed the mRNA levels of *RB1*, *DNM1L*, *RIPK3*, and *MLKL* in a whole genome analysis GEO cohort (Accession No. GSE93840) of primary human hepatocytes exposed to three xenobiotics (aflatoxin B1, amiodarone, and chlorpromazine) for 14 days^37^. The relative expression values of *RB1*, *DNM1L*, *RIPK3*, and *MLKL* were increased by these xenobiotics exposure (Supplementary Fig. S4). Further, *RB1* expression was positively correlated with *RIPK3*, *MLKL*, or *DNM1L* expression in these samples (*r* = 0.5879, 0.8523, or 0.8954, respectively, *P* < 0.05) (Fig. 4a–c). Proximity ligation assay (PLA) is a special immunoassay method that can be used to detect protein-protein interaction^38^. The PLA result showed that CdCl_2_ exposure enhanced the proximity of RB and MLKL (Fig. 4d, e). We also performed Co-IP assay with antibody against RB or RIPK3. We found that RIPK3 bound to RB in the mitochondrial fraction of liver tissues (Fig. 4f) and in the whole lysates of L02 cells treated with CdCl_2_ (Fig. 4g), and the MLKL, one component of the necrosome, was used as a positive control. Moreover, the binding between RB and Drp1 or RIPK3 were strengthened in L02 cells exposed to CdCl_2_ (Fig. 4h). These results indicated that CdCl_2_ enhanced RB binding to RIPK3 and promoting the formation of necrosome at mitochondria.

### RB, upregulated by CdCl_2_, translocates to mitochondria both in vivo and in vitro

**Fig. 5:**
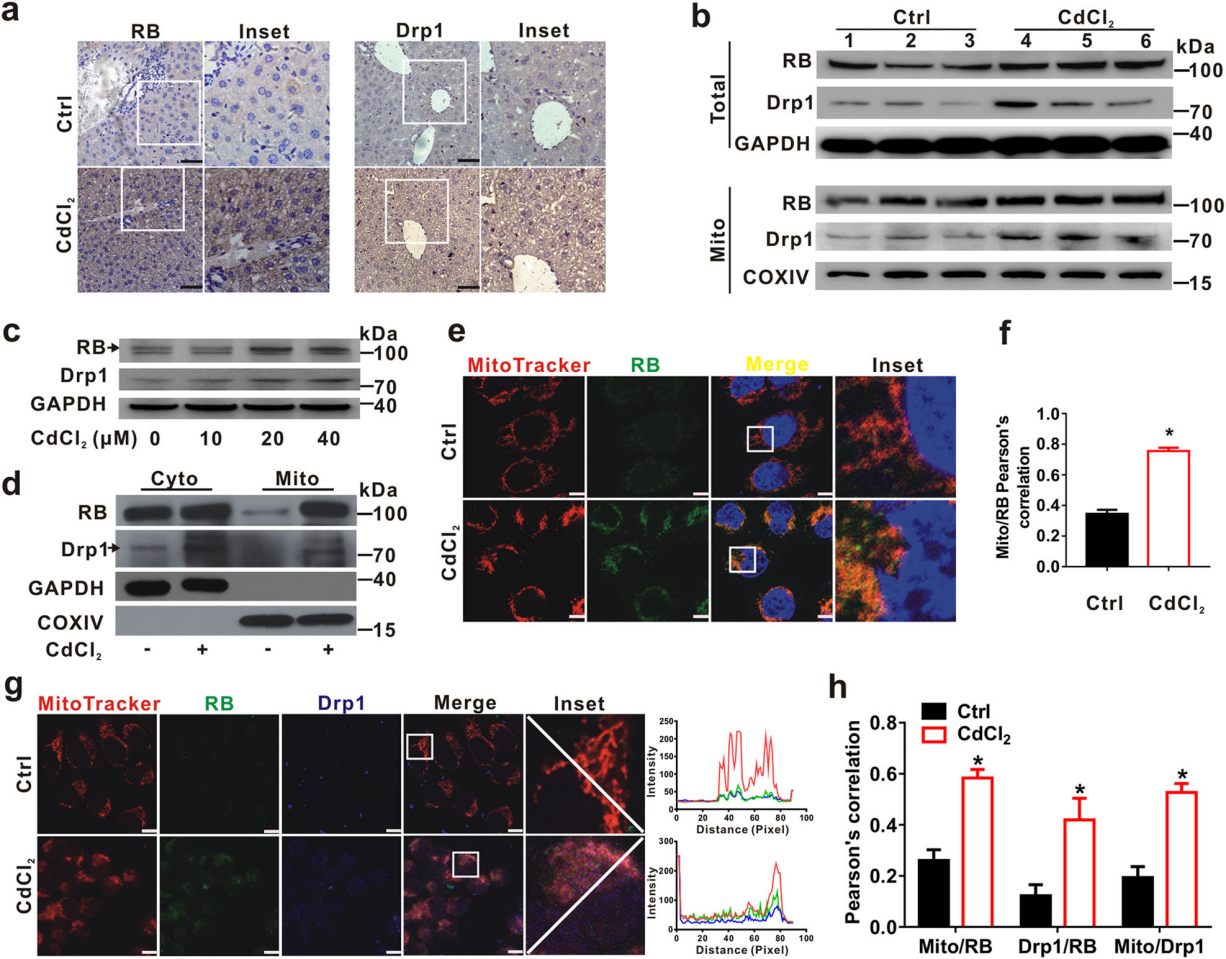
CdCl_2_ induces RB expression and its mitochondrial translocation both in vivo and in vitro. **a**, **b** The ICR mice were intraperitoneally injected with CdCl_2_ (1 mg/kg bw, *n* = 3) or physiological saline (Ctrl group, *n* = 3) for 7 days. **a** Liver slides were subjected to IHC using anti-RB and -Drp1 antibodies, respectively. The scale bar is 50 μm. **b** Whole cell lysates and mitochondrial fraction of liver tissues were prepared for Western blot with the indicated antibodies. **c** Western blot was applied to detect the expression levels of RB and Drp1 after CdCl_2_ (10, 20, and 40 μM) exposure to L02 cells for 6 h. (arrow denotes the specific band.) **d** Cytoplasmic (Cyto) and mitochondrial (Mito) fractions of L02 cells were prepared for Western blot to determine the expression of proteins as indicated. **e**–**h** L02 cells were treated with CdCl_2_ (20 μM) for 6 h. **e** Cells were subjected to IF staining with MitoTracker (red), anti-RB antibody (green), and DAPI (blue, staining nuclei). The scale bar is 10 μm. **f** Mito/RB signal overlap was analyzed with Pearson’s correlation. Red-green overlap sections were quantified by using ImagePro-plus 6.0. Ten fields of view were calculated for each group. **g** The images of L02 cells were labeled with MitoTracker (red) and immunostained with antibodies of anti-Drp1 (blue) and anti-RB (green). The scale bar is 10 μm. **h** Pearson’s correlation for the co-localization of Mito-RB, Drp1-RB, and Mito-Drp1 was shown in bar graphs quantified by using ImagePro-plus 6.0. Ten fields of view were calculated for each group. Data are expressed as the mean ± standard deviation (SD). *P* < 0.05, *significantly different from Ctrl

RB protein can directly target mitochondria to mediate apoptosis without relying on the classical nuclear transcriptional function^30^. Thus, we examined whether RB also affected necroptosis and hepatic injury through non-transcriptional regulation in liver of mice and L02 cells treated with CdCl_2_. The brown signals of RB and Drp1 expression were increased in the hepatocytes of liver tissues from mice treated with 1 mg/kg CdCl_2_ (Fig. 5a). Additionally, the levels of RB and Drp1 proteins were significantly augmented in CdCl_2_ group as detected using Western blot (Fig. 5b, upper panel). RB and Drp1 were also up-regulated in the mitochondrial fraction of liver tissues from mice exposed to CdCl_2_ (Fig. 5b, lower panel). In vitro, the expression levels of RB and Drp1 were also elevated by CdCl_2_ exposure in a concentration-dependent manner (Fig. 5c). As shown in Supplementary Fig. S5a, Western blot for cytoplasmic and nuclear fractions presented that RB mainly distributed in the cytoplasm of CdCl_2_ group. The mitochondrial distribution of Drp1 and RB were increased after CdCl_2_ exposure in cytoplasmic and mitochondrial fractions of L02 cells (Fig. 5d). Meanwhile, IF analysis revealed that RB fluorescence intensity was stronger in CdCl_2_ group than that in Ctrl group (Fig. 5e). The Pearson’s correlation of RB with mitochondria was significantly higher in CdCl_2_-treated cells (Fig. 5f). Consistently, CdCl_2_ increased the co-localization of mitochondrial RB and Drp1 (Fig. 5g, h) or p-Drp1^(Ser616)^ (Supplementary Fig. S5b). These results demonstrated that the up-regulation of RB and its translocation to mitochondria induced by CdCl_2_ might be through interaction with Drp1 both in vivo and in vitro.

### Inhibition of Drp1 counteracts excessive mitochondrial fission and RB translocation to mitochondria induced by CdCl_2_

**Fig. 6:**
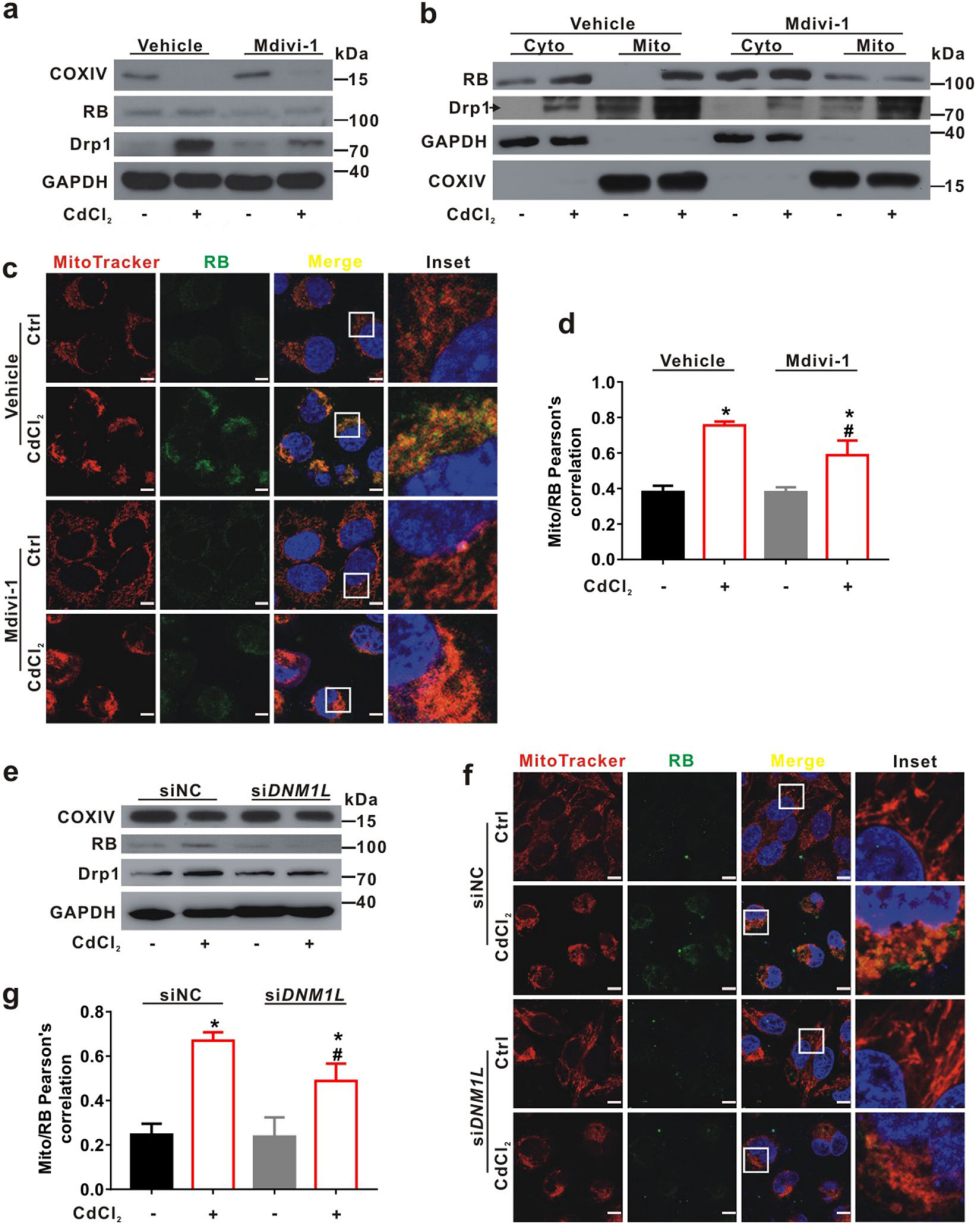
Inhibition of Drp1 counteracts mitochondrial fission and RB translocation to mitochondria induced by CdCl_2_ in L02 cells. **a**-**d** L02 cells were pretreated with Mdivi-1 (10 μM) for 30 min and then treated with CdCl_2_ (20 μM) for another 6 h. **a** Whole cell lysates and **b** cytoplasmic (Cyto) and mitochondrial (Mito) fractions of L02 cells in four groups were analyzed by Western blot with the indicated antibodies. **c** L02 cells were subjected to IF staining with MitoTracker (red) and anti-RB antibody (green). DAPI stained the nuclei. The scale bar is 10 μm. **d** Pearson’s correlation for the co-localization of mitochondria with RB is in bar graphs. Red-green overlap sections were quantified using ImagePro-plus 6.0. *P* < 0.05, *significantly different from Ctrl, ^#^significantly different from CdCl_2_ treatment. **e**-**g** Drp1 silencing with si*DNM1L* decreased Cd-induced mitochondrial fission and RB expression. siNC is the negative Ctrl of siRNA. **e** Whole cell lysates of L02 cells were analyzed by Western blot with the indicated antibodies. **f** L02 cells were subjected to IF staining with MitoTracker (red), anti-RB antibody (green), and DAPI (blue, staining nuclei). The scale bar is 10 μm. **g** Mito/RB signal with red-green overlap sections were quantified using ImagePro-plus 6.0. Ten fields of view were calculated for each group. Data are expressed as the mean ± standard deviation (SD). *P* < 0.05, *significantly different from Ctrl or siNC, ^#^significantly different from CdCl_2_ group

To elucidate whether mitochondrial distribution of RB is related to Drp1 in CdCl_2_-treated hepatocytes, Mdivi-1, an inhibitor of Drp1, was employed to pretreat L02 cells ahead of 30 min. As shown in Fig. 6a, the up-regulation of Drp1 and RB induced by CdCl_2_ was counteracted by Mdivi-1, while the level of COXIV was also restored. Western blot for mitochondrial fraction (Fig. 6b) and IF analysis (Fig. 6c, d) revealed that RB protein abundance and fluorescence signals were decreased in the mitochondria of L02 cells treated with a combination of CdCl_2_ and Mdivi-1. Moreover, siRNA for *DNM1L* (si*DNM1L*) transfection attenuated the CdCl_2_-induced mitochondrial fission and RB translocation to mitochondria (Fig. 6e–g). These results strongly suggested that Drp1 was involved in the regulation of RB expression and its mitochondrial translocation in hepatocytes treated with CdCl_2_.

### Drp1-RB axis regulates MQC and necroptosis in CdCl_2_-treated hepatocytes

**Fig. 7:**
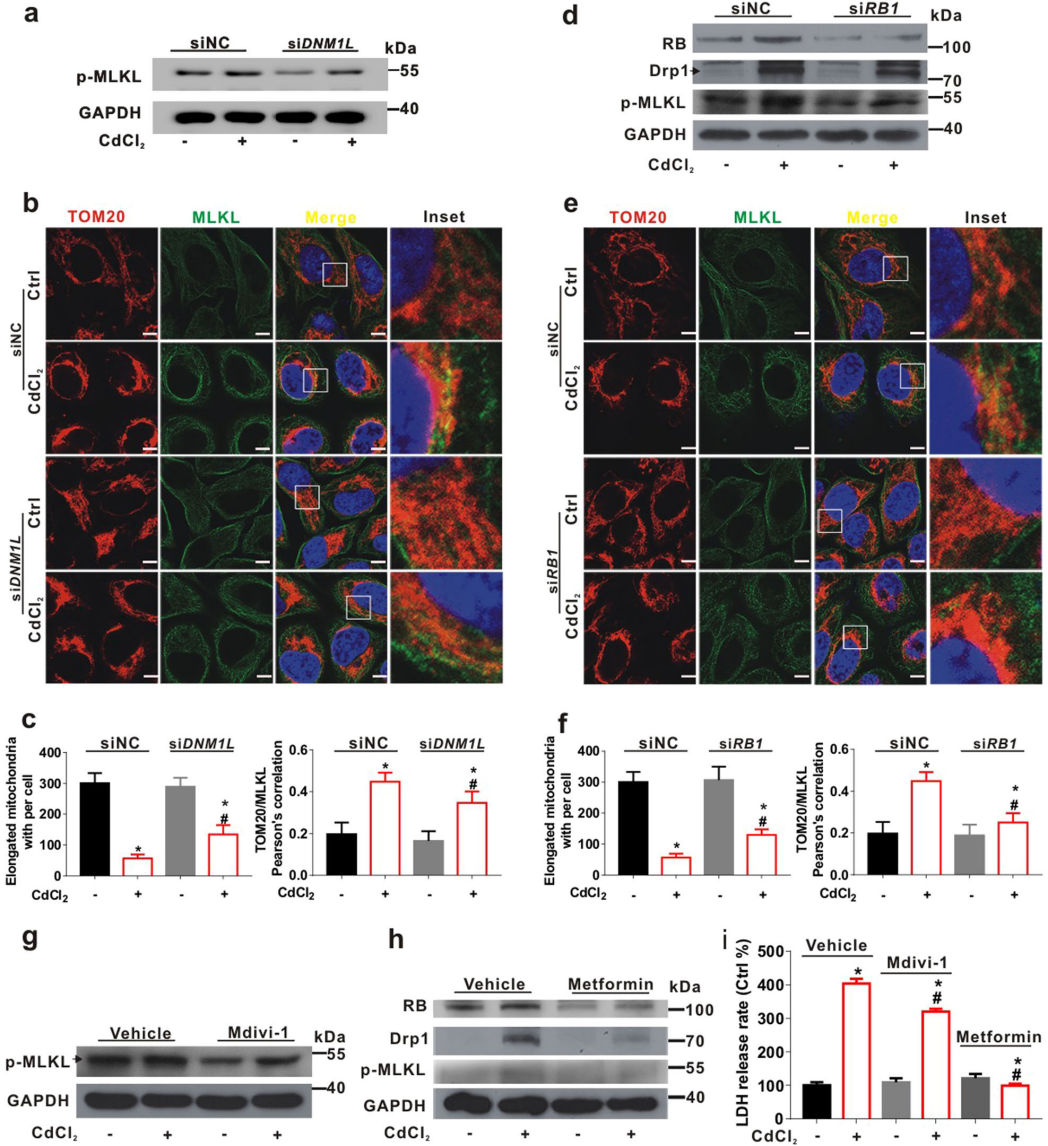
Drp1-RB axis regulates necroptosis triggered by CdCl_2_ in L02 cells. **a**-**f** Drp1 (**a**-**c**) or RB (**d**-**f**) silencing with si*DNM1L* or si*RB1* attenuated CdCl_2_-induced p-MLKL up-regulation in L02 cells. siNC is the negative Ctrl of siRNA. **a**, **d** Whole cell lysates of L02 cells of four groups were analyzed by Western blot with the indicated antibodies. **b, e** Confocal microscope images of L02 cells were immunostained with antibodies for anti-TOM20 (red) and anti-MLKL (green). The nuclei were labeled with DAPI (blue) (upper panels). The scale bar is 10 μm. **c**, **f** TOM20/MLKL signal with red-green overlap sections and the mitochondrial fragmentation counts were quantified using ImagePro-plus 6.0 (lower panels). **g**, **h** L02 cells were pretreated with Mdivi-1 (10 μM) (**g**) or metformin (**h**) for 30 min and treated with CdCl_2_ (20 μM) for another 6 h. Whole cell lysates of L02 cells were analyzed by Western blot with the indicated antibodies. **i** The LDH leakage was detected after Mdivi-1 or metformin combined with CdCl_2_ treatment. Data are expressed as the mean ± standard deviation (SD). Ten fields of view were calculated for each group. *P* < 0.05, *significantly different from Ctrl or siNC, ^#^significantly different from CdCl_2_ treatment

On the basis of above results, we employed siRNA for Drp1 (si*DNM1L* in Fig. 7a–c) and RB (si*RB1* in Fig. 7d–f) to investigate the regulatory role of Drp1-RB axis in MQC and necroptosis caused by CdCl_2_. The release rate of LDH was attenuated in CdCl_2_-treated L02 cells with si*DNM1L* or si*RB1* transfection (Supplementary Fig. S6). The elevation of p-MLKL induced by CdCl_2_ was inhibited by si*DNML1* (Fig. 7a). The IF images showed that mitochondrial fission and MLKL translocation to mitochondria induced by CdCl_2_ could be suppressed by si*DNM1L* (Fig. 7b). Further, the Pearson’s correlation of MLKL with mitochondria was significantly higher in CdCl_2_-treated cells with si*DNM1L* transfection (Fig. 7c). Similarly, knockdown of RB inhibited the elevation of p-MLKL, RB, and Drp1 in CdCl_2_-treated L02 cells (Fig. 7d). The mitochondrial fission and MLKL translocation to mitochondria induced by CdCl_2_ was suppressed by si*RB1* (Fig. 7e). Compared to CdCl_2_-treated cells, the Pearson’s correlation of MLKL with mitochondria was reduced in CdCl_2_-treated cells with si*RB1* transfection (Fig. 7f). TEM images also presented that si*RB1* attenuated the mitochondrial disorder caused by CdCl_2_ (Supplementary Fig. S7a). The rate of necroptotic cells was determined by PI staining using flow cytometry. We observed that si*RB1* inhibited the elevation of necroptotic cells rate caused by CdCl_2_ exposure (Supplementary Fig. S7b, c). Furthermore, inhibition of Drp1 with Mdivi-1 also reduced the expression of p-MLKL up-regulated by CdCl_2_ exposure (Fig. 7g). Metformin is widely used as an anti-diabetic drug, which can restore mitochondrial homeostasis by inhibiting mitochondrial fission^39,40^. As shown in Fig. 7h, the up-regulation of p-MLKL, RB, and Drp1 induced by CdCl_2_ were inhibited by metformin in L02 cells. Inhibition of Drp1 with Mdivi-1 or metformin also counteracted the increase of LDH release triggered by CdCl_2_ exposure (Fig. 7i). These results suggested that intervention of Drp1-RB axis could regulate MQC and inhibit necroptosis in CdCl_2_-exposed hepatocytes.

### Metformin regulates Drp1-RB axis and rescues CdCl_2_-induced hepatic necroptosis and injury in vivo

**Fig. 8:**
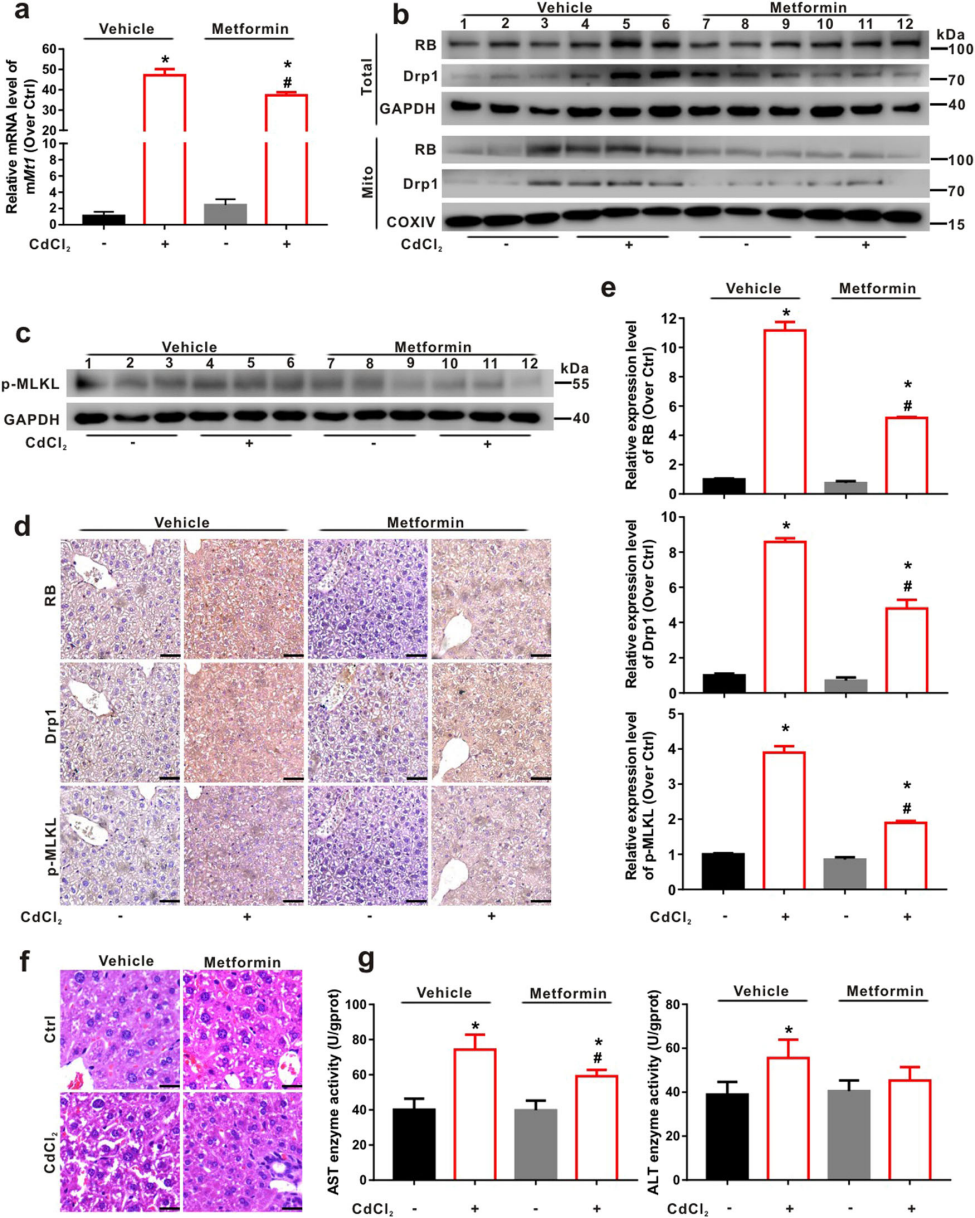
Metformin regulates Drp1-RB axis and rescues CdCl_2_-induced hepatic necroptosis and liver injury in mice. The ICR male mice were intragastrically administrated with metformin (100 mg/kg bw) and intraperitoneally injected with CdCl_2_ (1 mg/kg bw) every day for one week. Vehicle was physiological saline as a solvent control. *n* = 3. **a** m*Mt1* gene expression was detected using qRT-PCR. m*Gapdh* was used as the reference gene. **b** Total lysates and mitochondrial fraction of liver tissues were prepared for SDS-PAGE with the indicated antibodies. **c** p-MLKL protein was evaluated by using Western blot. **d**, **e** IHC assays with serial sections of liver were observed for tissue distribution of RB, Drp1, and p-MLKL with the indicated antibodies. **d** The representative IHC images were shown. **e** The brown intensity of IHC images was quantified using ImagePro-plus 6.0. **f** HE staining of sections of liver was shown. The scale bar is 50 μm. **g** The activities of AST and ALT were detected after CdCl_2_ exposure with/without metformin. Data are expressed as the mean ± standard deviation (SD). *P* < 0.05, *significantly different from Ctrl group, ^#^significantly different from CdCl_2_ treatment

We further applied metformin to verify whether it can inhibit hepatic necroptosis and the related liver injury in CdCl_2_-adminstered male mice. The high expression of metallothioneins (MTs) is a typical biomarker for exposure of metals, including Cd^41^. The mRNA level of m*Mt1* was induced in the liver of mice intraperitoneally injected with CdCl_2_ (1 mg/kg) for one week (Fig. 8a). Western blot result presented that metformin blocked the elevation of RB and Drp1 caused by CdCl_2_ in the total lysates and the mitochondrial fraction of liver tissues (Fig. 8b). As shown in Fig. 8c, metformin inhibited the up-regulation of liver p-MLKL caused by CdCl_2_. IHC analysis also indicated that the brown signals of RB, Drp1, and p-MLKL expression increased by CdCl_2_ treatment were inhibited by metformin (Fig. 8d, e). Meanwhile, metformin significantly attenuated CdCl_2_-caused hepatic cord rupture and vacuolization in liver as detected using HE staining (Fig. 8f). The activities of AST and ALT were also significantly increased in liver lysates of mice treated with CdCl_2_. On the other hand, metformin pretreatment partially alleviated the elevation of liver enzymes activities, especially AST caused by CdCl_2_ administration (Fig. 8g). To further assessed whether metformin inhibits CdCl_2_-induced hepatic necroptosis and the related liver injury in female mice, as shown in Figs. S8, S9, similar results were obtained in vivo (in male and female mice). Taken together, these findings demonstrate that CdCl_2_-induced hepatic necroptosis and liver injury could be rescued by the inhibition of Drp1-RB axis.

## Discussion

The hepatotoxicity of Cd is mainly manifested as hepatic dysfunction, necroinflammation, lipid accumulation, and fibrosis^42,43^. Previous studies show that Cd-induced hepatotoxicity is involved in apoptosis, calcium homeostasis, oxidative stress, and inflammation^22,44^. In the present study, we found a new mechanism of CdCl_2_-induced hepatotoxicity associated with necroptosis and its regulatory mechanism of Drp1-RB axis. Our study exhibited that CdCl_2_ caused the hepatic necroptosis and related liver injury both in vivo and in vitro. Meanwhile, MQC disorder with mitochondrial dysfunction and excessive fragmentation was proved to be mediated by mitochondrial Drp1 up-regulation, which led to necroptosis in CdCl_2_-treated mice and hepatic cells. Besides, RB protein was induced and translocated to mitochondria to interact with Drp1 after CdCl_2_ exposure, which promoted the formation of necrosome harbored at mitochondria. Furthermore, metformin was employed to inhibit Drp1-RB axis, which attenuated hepatic necroptosis and related liver injury triggered by CdCl_2_ exposure in mice. Most importantly, we, for the first time, demonstrated that CdCl_2_-induced necroptosis and hepatotoxicity were regulated by Drp1-RB axis and intervention of this axis would be a potential valuable strategy to prevent xenobiotics-induced necroptosis and hepatotoxicity.

MQC is tightly linked to CdCl_2_-caused hepatic injury^45,46^, which was reported to be regulated by mitochondrial Ca^2+^ and autophagy previously^22^. Our results also validated that CdCl_2_ induced excessive mitochondrial fssion and mitoROS, loss of Δψm, and decrease of ATP production. Moreover, Pi et al reported that Drp1 mediated-mitochondrial fission and other mitochondrial damages^44^. Consistently, we also demonstrated that Drp1 was up-regulated by CdCl_2_ exposure and blocking Drp1 rescued mitochondrial dysfunction, hepatic necroptosis, and liver injury. Moreover, we illuminated that Drp1 up-regulation was related with the MQC disorder contributed to CdCl_2_-induced necroptosis and hepatotoxicity.

As one of the most common heavy metal pollutants, CdCl_2_ exposure can result in necrosis-like cell death in a variety of cells^25,47^. For example, CdCl_2_ exposure lead to necroptosis of human lung L929 cells^47^. Necroptosis is characterized as disruption of the plasma and intracellular membranes, which is mediated by MLKL phosphorylation and its membrane translocation^25^. We identified that CdCl_2_ exposure caused mitochondrial swelling and cell membrane rupture by TEM and LDH leakage assay. Wang et al. also found that membrane translocation of MLKL was regulated by RIPK3^25^. In our study, both RIPK3 and p-MLKL were increased in CdCl_2_-exposed hepatocytes. CdCl_2_ also induced mitochondrial translocation of MLKL and necrosome formation harbored at mitochondria. Mitochondrial localized MLKL can bind to cardiolipin and lead to membrane disruption, which is the key to necroptosis^25^. Herein, we deduced that necrosome translocation to mitochondria was involved in the regulation of MQC disorder caused by CdCl_2_ in hepatocytes.

Many studies have presented that necroptosis is inhibited in tumor and epithelial cells through down-regulating Drp1^8,48^. In this study, we discovered that inhibition of Drp1 was able to block mitochondrial translocation of MLKL and its phosphorylation caused by CdCl_2_. Meanwhile, Drp1 has been proven to be a downstream molecule of RIPK1-RIPK3 complex. For instance, Wang et al. reported that RIPK1-RIPK3 induced Drp1 expression and translocation to mitochondria in macrophages triggered by RNA viruses^2^. Besides, mitochondrial translocation of Drp1 was raised by acetaminophen treatment in hepatocytes, which was impaired in RIPK3 knockout mice^49^. In the present study, we found that Drp1 directly bound to RIPK3 in mitochondrial fraction, which was further enhanced by CdCl_2_ exposure. Thus, these studies demonstrate that Drp1 regulates xenobiotics-induced necroptosis through interacting with RIPK3 at mitochondria.

In the present study, RB was proved to bind to the necrosome. We further discovered that RB interference inhibited the up-regulation of p-MLKL expression, mitochondrial translocation of MLKL, and increased of necroptotic cell rate caused by CdCl_2_ exposure. Moreover, there are increasing lines of evidence that RB protein translocates to mitochondria and regulates MQC^50–53^. For example, RB knockdown also increases mitochondrial fatty acid oxidation in breast cancer cells^51^. In *Drosophila*, RB homologue (Rbf1) also increases mitochondrial fragmentation through up-regulation of Drp1^52^. Similar to p53, RB is one of tumor suppressor and its function is divided into transcriptional-dependent and -independent regulations^30^. Drp1 inhibition by a peptide inhibitor P110 diminishes mitochondrial translocation of p53 in a Parkinson’s disease mouse model^54^. Drp1 interacts and stabilizes p53 at mitochondria to induce necrosis under oxidative stress conditions^55^. In the current study, we proved that Drp1 could directly bind to RB at mitochondria. We further demonstrated that CdCl_2_ induced RB expression and its translocation to mitochondria, which was also counteracted by down-regulation of Drp1. These researches indicated that RB mitochondrial translocation directly interact with Drp1, resulting in MQC disorder and necroptosis in hepatocytes triggered by CdCl_2_ exposure.

It is urgent to seek for an effective intervention approach to inhibit necroptosis-related diseases in clinic^3^. Sorafenib, a drug approved for hepatocellular carcinoma at late stage by Food and Drug Administration (USA), has been found to target RIPK1-RIPK3 pathway and protect mice from inflammation^56^. Likely, metformin, as a drug for anti-type 2 diabetes and cancer^57,58^, had an inhibitory effect on necroptosis through regulating Drp1-RB axis in our study. We found that blocking Drp1 with metformin rescued CdCl_2_-induced hepatic necroptosis and liver injury through inhibiting p-MLKL up-regulation, and the expression and mitochondrial translocation of RB protein, although the inhibitory effect of metformin on necroptosis may be indirect^40,59^. Taken together, Drp1-RB axis might become a potential target for drug prevention of necroptosis-associated liver diseases in the future.

In conclusion, we have demonstrated that activation of mitochondrial Drp1 enhances mitochondrial translocation of RB in hepatocytes induced by CdCl_2_ both in vivo and in vitro (Fig. S10). The mitochondrial RB directly interacts with Drp1 and participates in the formation of MLKL-associated necrosome, regulating hepatocyte’s necroptosis and liver injury caused by CdCl_2_ exposure. Moreover, interventions of Drp1-RB axis with gene knockdown or pharmaceutical inhibitors alleviate CdCl_2_-induced necroptosis and hepatotoxicity. Our work indicated that the mitochondrial Drp1-RB axis was a potential intervention target and one of candidate markers of liver injury and associated diseases related to xenobiotics exposure.

## Materials and methods

### Cell culture and treatments

The human immortalized hepatic L02 cell line was cultured in RPMI 1640 medium (Hyclone, Logan, UT, USA) containing 10% fetal bovine serum (Life, New York, NY, USA) and 1% (v/v) penicillin/streptomycin (Life) in a 5% CO_2_ humidified atmosphere at 37 °C. Cells were treated with CdCl_2_ (Sigma, St. Louis, MO, USA) at different concentrations (10, 20, and 40 μM) for 6 h or with 20 μM CdCl_2_ for different durations (3, 6, and 12 h).

### Animal studies

According to our previous experiment^33^, ICR mice (male and female, 10 ~ 12-weeks old) were divided into control (Ctrl) group (0.9% physiological saline) and CdCl_2_ exposure group (1 mg/kg body weight) and raised in a specific pathogen-free facility of Xiamen University. Mice were intraperitoneally injected with 0.9% physiological saline or CdCl_2_ for seven days. The body weight was recorded every day. After exposure, mice were sacrificed and their serum and livers were collected, frozen or fixed for following experiments. As for the intervention experiment, mice were divided into four groups: control (Ctrl) group, CdCl_2_ exposure group, metformin (Met) group (100 mg/kg bw) pretreatment control, and metformin pretreatment with CdCl_2_ exposure groups. Both metformin and CdCl_2_ were dissolved in physiological saline. The mice were intragastrically administrated with metformin and intraperitoneally injected with CdCl_2_ every day for one week. All experiments were performed in accordance with the guidelines and approved by the Animal Ethical Committee of Xiamen University.

### Reagents

Benzyloxycarbonyl-Val-Ala-Asp fluoromethylketone (z-VAD) was from EnzoLife Sciences (Farmingdale, NY, USA). TNF-α were purchased from R&D systems (Minneapolis, MI, USA). Mdivi-1 and necrosulfonamide (NSA) were purchased from Selleck (Shanghai, China). Alexa Fluor-405 Goat anti-rabbit IgG (H + L) and Alexa Fluor-488 Goat anti-mouse IgG (H + L) secondary antibodies (P0195d and A0428) and anti-COXIV (AC610) antibody were purchased from Beyotime (Shanghai, China). Horseradish peroxidase (HRP)-conjugated secondary antibodies against rabbit or mouse were from Thermo (Waltham, MA, USA). Propidium iodide (PI), 4',6-diamidino-2-phenylindole (DAPI), dimethyl sulphoxide (DMSO), and rabbit anti-MLKL antibody (M6697) were from Sigma. Rabbit anti-RIPK3 (ab72106), rabbit anti-p-MLKL (phospho Ser358) (ab187091), and mouse anti-Mfn2 (ab56889) antibodies were purchased from Abcam (Cambridge, MA, USA). Mouse anti-RIPK1 (610459) and anti-TOM20 antibodies (612278) were obtained from BD Biosciences (San Jose, CA, USA). Mouse anti-RB (9309), rabbit anti-p-RB (phospho Ser807/811) (8516), and anti-p-Drp1 (phospho Ser616) (3455 S) antibodies were purchased from Cell Signaling Technology (Danvers, MA, USA). Rabbit anti-Drp1 (RLT1414) was from Ruiying (Suzhou, China). The antibody against GAPDH (6C5) (mb001) was from Kangchen (Shanghai, China).

### Cell death assay

L02 cells were trypsinized and suspended into single cells and incubated with 5 μg/mL PI in PBS for 20 min. PI positive cells (indicating cell death) were analyzed by flow cytometry (Beckman, Pasadena, CA, USA) with excitation/emission wavelength of 561/617 nm approximately.

### Lactate dehydrogenase (LDH) assay

The LDH leakage in supernatant of cell culture medium was detected for cellular membrane damage. The operation was according to the instructions of LDH test kit (Beyotime) with slight modification. Briefly, after treatment, the 96-cell culture plate was centrifuged at 400 × *g* for 5 min. The cell-free supernatant (120 μL) from each well was collected. Then, 60 μL of LDH test solution was added and incubation for 30 min at room temperature in the dark. The absorbance (490 nm) was measured at 37 °C with a multifunctional microplate reader (BMG LRBTECH CLARIOstar, Offenburg, Germany).

### Transmission electron microscope (TEM) assay

L02 cells were collected and fixed in 2.5% gluteraldehyde at 4 °C for 2 h and postfixed with 1.5% osmium tetroxide for 2 h. Then the cell pellets were dehydrated through acetone and embedded in resin. The pieces were sectioned at 70 nm thickness and stained with lead citrate after hardening. The ultrastructure of cells was observed by TEM (FEI Tecnai 20, Hillsboro, OR, USA) described as our previous study^60^.

### Immunofluorescence (IF) assay

L02 cells with different treatment were labeled for mitochondria and intracellular Cd^2+^ with MitoTracker Deep Red (Life) and Leadmium™ Green AM Dye (Cd^2+^ probe; Thermo) for 30 min at 37 °C, respectively. Then, cells were fixed in 4% paraformaldehyde (pH 7.4) for 30 min at room temperature. Blocking buffer (1× PBS, 1% bovine serum albumin, 0.3% Triton™ X-100) was applied to the sample for 45 min. After incubating with the primary antibodies (anti-RIPK1, -RIPK3, -RB, -Drp1, -TOM20, -MLKL, or -p-MLKL) at 4 °C overnight, the samples were incubated with goat anti-mouse/rabbit IgG fluorescent secondary antibody for 1 h and counterstained with DAPI. The whole process was protected from light. Finally, stained cells were visualized using a confocal microscope (Zeiss LSM 780, Carl Zeiss, Jena, Germany) equipped with ×63 oil objective. Quantification of protein localization on mitochondria was analyzed by ImagePro-plus 6.0.

### Proximity ligation assay (PLA)

PLA is a special immunoassay method that can be used to detect protein and protein interaction. The specific method was mentioned in the previous article^61^. Briefly, L02 cells were plated on sterile slides in twelve-well plates for 24 h before administration. After being fixed and permeabilized, the slides were incubated in Duolink II solution for 1 h. The samples were incubated with primary antibodies: anti-RB antibody (1:500) and anti-RIPK3 antibody (1:400) at 4 °C overnight, followed by incubation of anti-mouse or anti-rabbit Duolink PLA probe (Sigma). Before counterstained with DAPI, the sample were incubated with the ligase (1:40) for 30 min and with the polymerase (1:80) for another 100 min at 37 °C. Image observation and processing were consistent with IF assay.

### Small interfering RNA (siRNA) transfection

Cells were seeded at six-well plates close to 60% confluence and transfected with a pool of siRNA sequences mixed with Lipofectamine® 2000 (Life) at a final concentration of 50 nM for 10 h. The cells were recovered for 24 h after removal of transfection agents and then treated with various concentrations of CdCl_2_. Si*RB1*, si*DNM1L*, and siNC were purchased from Ribobio Company (Guangzhou, China).

### Isolation of cytoplasmic (Cyto) and mitochondrial (Mito) fractions

The Cyto and Mito fractionation kit (EnzoLife) was used to separate the cellular components. In brief, after washing twice with cold PBS, at least 5 × 10^7^ cells of each group were collected and then centrifuged at 600 × *g* at 4 °C for 5 min. The pellets were resuspended in mitochondria isolation buffer containing dithiothreitol and protease inhibitors and placed on the ice for 15 min. The mixtures were stirred with a glass homogenizer and centrifuged at 700 × *g* for 10 min. The supernatants were gathered and centrifuged at 10,000 × *g* at 4 °C for 30 min. This step was the key to achieve mitochondria and cytoplasm separation. The pellet was mitochondria, which was resuspended in mitochondrial lysate buffer and vortex oscillated for 15 s. Cyto and Mito proteins were quantified by the bicinchoninic acid protein assay reagent (Beyotime) and were used for Western blot. COXIV or GAPDH was used as loading control for the Mito or Cyto fractions, respectively.

### Co-immunoprecipitation (Co-IP)

For RB or necrosome immunoprecipitation, cells were seeded in 10 cm tissue culture dish, grew to reach 80% confluence and then were treated with CdCl_2_ (20 μM) for 6 h. After treatment, cells were rinsed twice with ice-cold PBS and lysed with Nonidet P-40 lysis buffer (Sigma). SureBeads (100 μL) (BioRad, Hercules, CA, USA) were transferred into 1.5 mL Eppendorf tube. PBST (PBS plus 0.1% Tween 20) washed three times and discarded supernatant by magnetic separator. Then 200 μL IP antibodies were adhered to SureBeads in shaker for 20 min. Cell lysates were subjected to IP with mouse anti-RIPK3 or anti-RB beads at room temperature for 1 h. Then, the beads were washed three times in PBST. The proteins were eluted by 1× SDS buffer and analyzed by Western blot. Mitochondrial fraction was extracted from fresh mouse liver tissues using the same procedure as for cells. Isolated mitochondrial fraction was used for Co-IP experiment with the same method described as above.

### Western blot

Cells or liver tissues were lysed in 1 × sodium dodecyl sulfate-polyacrylamide gel (SDS-PAGE) loading buffer or radio immunoprecipitation assay buffer (Beyotime) with 1% protease inhibitors and phosphatase inhibitor cocktail. Samples were loaded in SDS lysis buffer (10% SDS, 0.25 M Tris-HCl at pH 6.8, 50% glycerol, 1% β-mercaptoethanol, bromophenol blue), separated by SDS-PAGE and then transferred to polyvinylidene fluoride membranes (Millipore, Boston, MA, USA). After blocking with 5% skimmed milk for 1 h, the membranes were incubated with the primary antibodies at 4 °C overnight and then with the HRP-labeled secondary antibody for 1 h. Western blot bands were visualized with an enhanced chemiluminescence kit (Advansta, Menlo Park, CA, USA).

### Histology and immunohistochemistry (IHC) staining

For qualitative analysis of hepatic histology, liver tissues were fixed in 4% paraformaldehyde (pH 7.4) and embedded in paraffin. Sections of 4–5 μm were mounted microscope slides. The slides were dewaxed by xylene and rehydrated by gradient of ethanol for hematoxylin and eosin (HE) staining and immunohistochemistry (IHC) analysis. As for histology, after dewaxed, the sections were rehydrated, stained with HE, and subsequently subjected to pathological assessment using an optical inverted microscope (TS-100, Nikon, Tokyo, Japan). As for IHC, after incubation with 0.01 M sodium citrate (pH 6.0) for antigen retrieval and endogenous peroxidase blocking solution, the slides were incubated with 50 μL non immune animal serum for 30 min to block nonspecific proteins. Then, the slides were incubated with specific primary antibody at 4 °C overnight and biotinylated secondary antibody for 30 min. Slides were incubated with streptomycin biotin-peroxidase for 45 min and incubated with peroxidase substrate diaminobenzidine, following with hematoxylin counterstaining of nuclei. Moreover, the slices were dehydrated by gradient dehydration of ethanol, permeated in xylene and mounted by resin. Ultimately, images were captured by inverted microscope (Nikon) and analyzed by ImagePro-plus 6.0 software.

### Quantitative real-time polymerase chain reaction (qRT-PCR)

Total RNA was isolated using TRIzol reagent (TaKaRa, Otsu, Japan) and reverse-transcribed using reverse transcription reagents (TaKaRa). QRT-PCR was performed according to previous study^33^. The following primers were used: m*Gapdh* (5′-TTG ATG GCA ACA ATC TCC AC-3′ and 5′-CGT CCC GTA GAC AAA ATG GT-3′), m*Mt1* (5′-CTC TAA GCG TCA CCA CGA CTT C-3′ and 5′-CGT CAC ATC AGG CAC AGC AC-3′).

### The assays of aspartate aminotransferase (AST) and alanine aminotransferase (ALT) activities

AST and ALT were used as two biochemical markers for hepatic damage, and were determined using commercial kits (Jiancheng, Nanjing, China). Briefly, liver tissues were homogenized on ice bath and centrifuged to collect the supernatants. Then, the corresponding operation was performed according to the instructions. The optical density at 510 nm was detected by multifunctional microplate reader.

### Statistics

Statistical analyses used the statistical package for social sciences (SPSS) version 16.0 (SPSS, Chicago, IL, USA). All data were expressed as the mean ± SD at least three independent experiments. Statistical analyses were performed by using one-way analysis of variance (ANOVA) and Student’s *t*-test. Pearson’s correlation analysis was conducted for variables correlation. *P* < 0.05 (two tailed) was considered to have statistical significance.

## Supplementary information

Supplementary materials
