# Supplementary material for: Drp1 and RB interaction to mediate mitochondria-dependent necroptosis induced by cadmium in hepatocytes
Source: Cell Death Dis. 2019 Jul 8;10(7):523. doi: 10.1038/s41419-019-1730-y (PMC6614419; doi:10.1038/s41419-019-1730-y)
Supplement: Supplementary file 1 — Supplementary materials [file 41419_2019_1730_MOESM1_ESM.docx]

**Supplementary materials**

**Supplementary Fig. S1:**


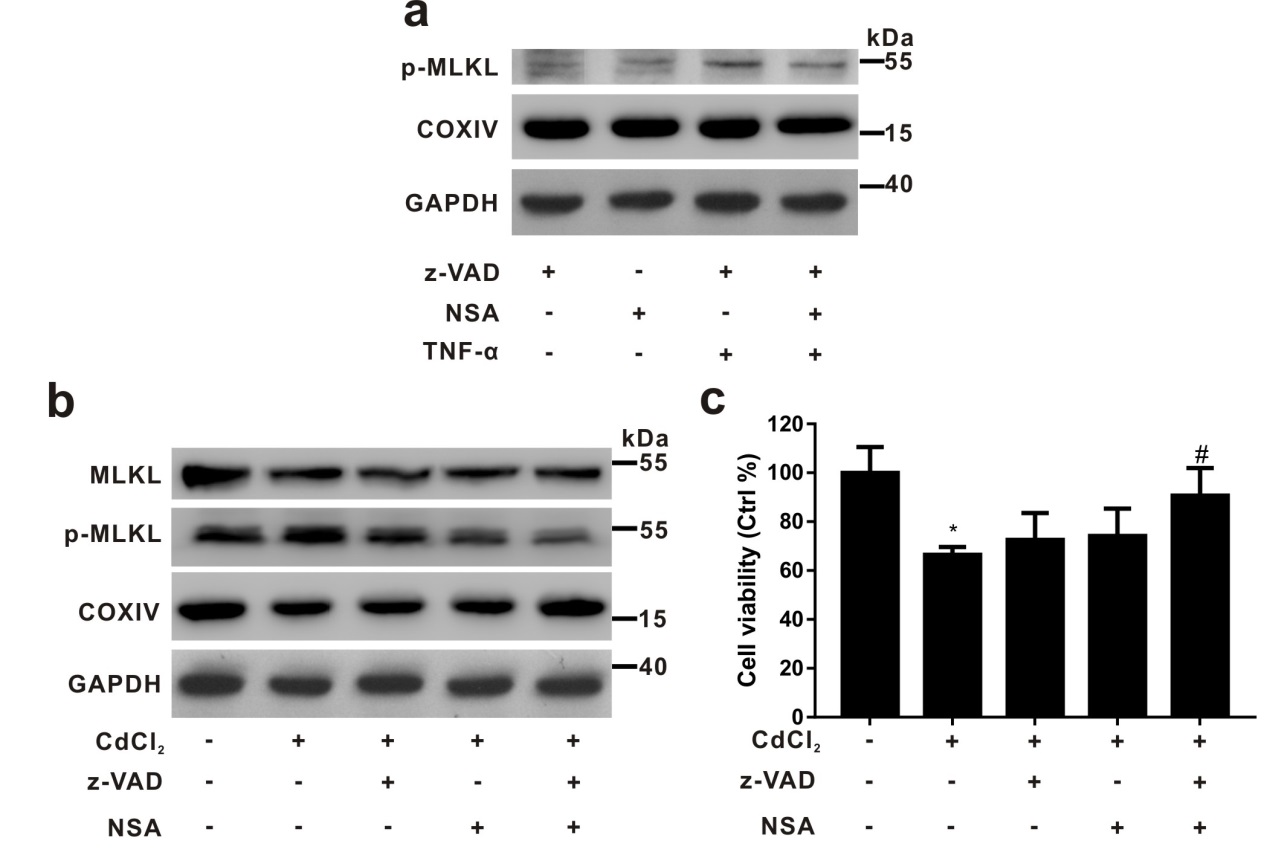


**Supplementary Fig. S1** **Necroptosis is induced by CdCl_2_ and inhibited by NSA *in vitro*. a** Positive model of necroptosis in L02 cells with different treatment. **b-c** L02 cells were treated with CdCl_2_ (20 mM) and/or z-VAD (20 mM) and/or NSA (1 mM) for 6 h, which was evaluated by Western blot and (3-(4,5-dimethylthiazol-2-yl)-2,5-diphenyltetrazolium) bromide (MTT) assay. Data are expressed as the mean ± standard deviation (SD). n = 4. *P* < 0.05, * significantly different from Ctrl group, ^#^ significantly different from CdCl_2_ group.

**Supplementary Fig. S2:**


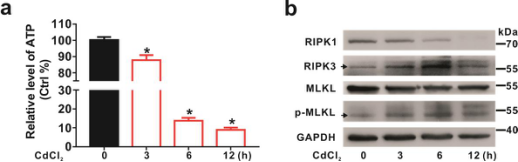


**Supplementary Fig.S2 CdCl_2_ exposure results in the decrease of ATP and the increase of RIPK3 and p-MLKL proteins in hepatic L02 cells in a time-dependent manner. a** ATP level was detected using an ATP Assay Kit. Data are expressed as the mean ± standard deviation (SD). n = 4. *P* < 0.05, * significantly different from Ctrl group. **b** The levels of necroptosis-related proteins (RIPK1, RIPK3, MLKL, and p-MLKL) were analyzed by Western blot with the indicated antibodies. Arrow denotes the specific band.

**Supplementary Fig. S3:**


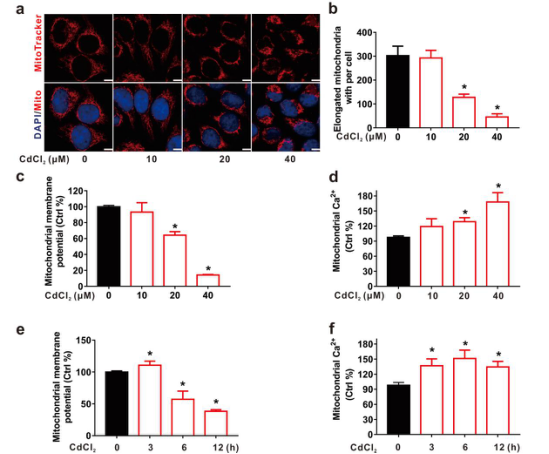


**Supplementary Fig. S3** **CdCl_2_ exposure disorders mitochondrial quality control (MQC) function in L02 cells. a-b** Mitochondria were stained with MitoTracker after exposure CdCl_2_ for 6 h. **a** The representative images with confocal microscopy were shown. The scale bar is 10 mm. **b** The mitochondrial morphology was analyzed. Cells with enlongated mitochondria were evaluated by using ImagePro-plus 6.0. Ten fields of view were calculated for each group. **c-f** The mitochondrial membrane potential (Δψm) and mitochondrial Ca^2+^ in CdCl_2_-treated L02 cells were measured by JC-1 staining assay and mitochondrial Ca^2+^ probe (Rhod-2 AM). **c, e** Δψm was dropped in concentration-and time-dependent manner. **d, f** Mitochondrial Ca^2+^ was increased by CdCl_2_ in concentration-and time-dependent manner. Data are expressed as the mean ± standard deviation (SD). n = 4. *P* < 0.05, * significantly different from Ctrl group.

**Supplementary Fig. S4:**


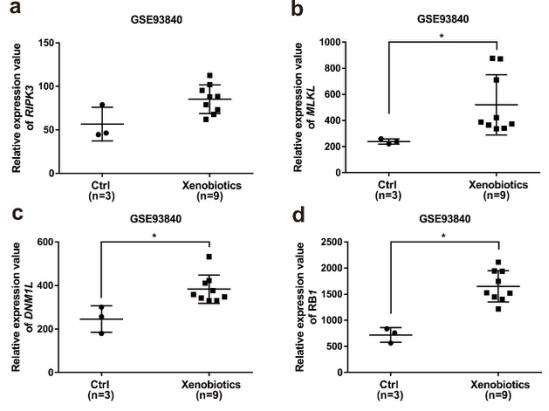


**Supplementary Fig. S4 The relative expression values of *RIPK3* (a), *MLKL* (b), *DNM1L* (c), and *RB1* (d) were obtained from GEO database.** The relative expression values in [primary human hepatocytes exposed to three xenobiotics](https://www.ncbi.nlm.nih.gov/geo/query/acc.cgi?acc=GSE31286) (aflatoxin B1, amiodarone, and chlorpromazine) for 14 days from NCBI, GEO database (Accession No. GSE93840) was calculated. Data are expressed as the mean ± standard deviation (SD). *P* < 0.05, * significantly different from Ctrl group.

**Supplementary Fig. S5:**


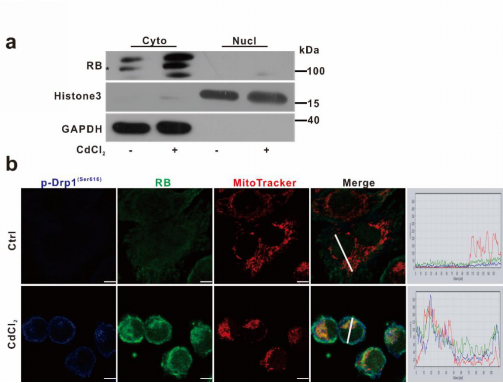


**Supplementary Fig. S5** **The intracellular localization of RB was detected after CdCl_2_ exposure in L02 cells.** **a** Cytoplasmic (Cyto) and nuclear (Nucl) fractions were analyzed by Western blot with the indicated antibodies. (* denotes specific band.) **b** Confocal microscopy images of L02 cells immunostained with anti-p-Drp1^(Ser616)^ (blue), anti-RB (green) antibodies, and MitoTracker (red). The profiles of representative lines trace the intensities of p-Drp1^(Ser616)^ and RB signals along with MitoTracker. Fluorescence curve was analyzed with line intensity profile by Zen 2012 software. The scale bar is 10 mm.

**Supplementary Fig. S6:**


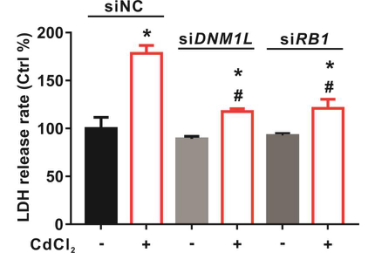


**Supplementary Fig. S6** ***DNM1L* or *RB1* silencing decreased LDH leakage induced by CdCl_2_ in L02 cells.** Drp1 or RB silencing with si*DNM1L* or si*RB1* attenuated CdCl_2_-triggered LDH release up-regulation in L02 cells. siNC is the negative Ctrl of siRNA. Data are expressed as the mean ± standard deviation (SD). n = 4. *P* < 0.05, * significantly different from siNC, ^#^ significantly different from CdCl_2_ treatment.

**Supplementary Fig. S7:**


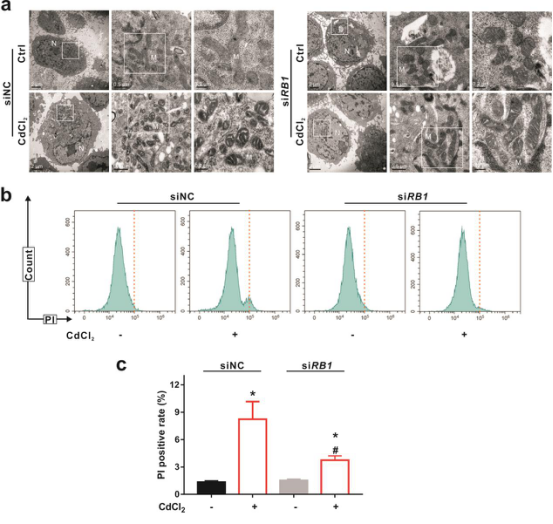


**Supplementary Fig. S7 RB** **knockdown inhibits CdCl_2_-induced necroptosis in L02 cells. a** Ultrastructure structure was observed by TEM after exposure CdCl_2_ with si*RB1* in L02 cells. The scale bar is 2 μm, 0.5 μm, and 0.2 μm respectively. M, mitochondrion; N, nucleus. **b** *RB1* silencing with si*RB1* decreased PI positive rate compared with that in the CdCl_2_ group. siNC is the negative Ctrl of siRNA. L02 cells stained with PI were detected by flow cytometry analysis. **c** Quantification of PI positive rate was shown in bar charts. Data are expressed as the mean ± standard deviation (SD). n = 3. *P* < 0.05, * significantly different from siNC, ^#^ significantly different from CdCl_2_ treatment.

**Supplementary Fig. S8:**

**
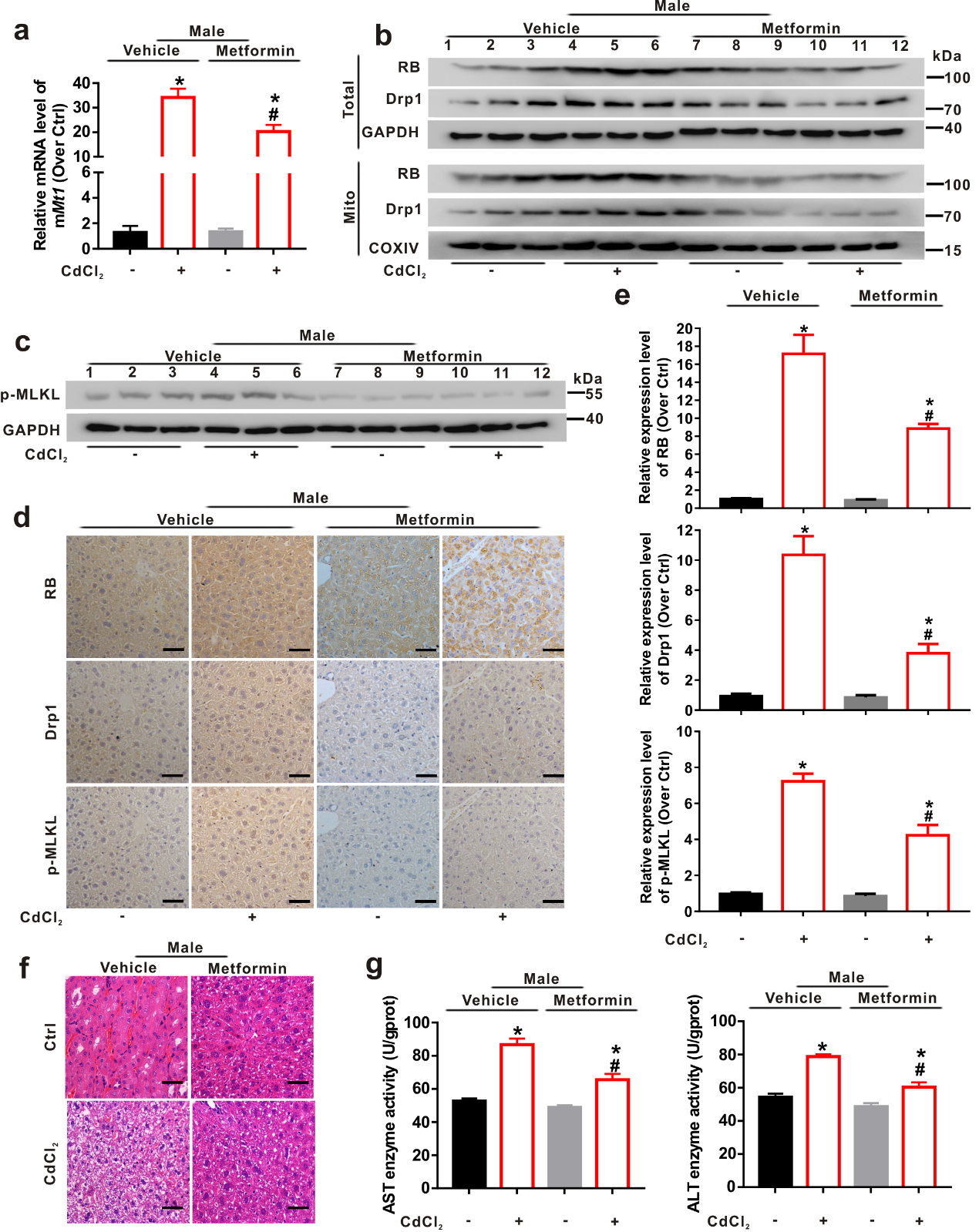
**

**Supplementary Fig. S8 Metformin regulates Drp1-RB axis and rescues CdCl_2_-induced hepatic necroptosis and liver injury in male mice.** The ICR male mice were intragastrically administrated with metformin (100 mg/kg bw) and intraperitoneally injected with CdCl_2_ (1 mg/kg bw) every day for one week. Vehicle was physiological saline as a solvent control. n = 3. **a** The mRNA level of m*Mt1* gene was detected by using qRT-PCR. m*Gapdh* was used as the reference gene. **b** Total lysates and mitochondrial fraction of liver tissues were prepared for SDS-PAGE with the indicated antibodies, the expression levels of RB, Drp1, and p-MLKL were evaluated by using Western blot. **c** p-MLKL protein was evaluated by using Western blot. **d-e** IHC assays with serial sections of liver were observed for tissue distribution of RB, Drp1, and p-MLKL with the indicated antibodies. **d** The representative IHC images were shown. **e** The relative expression levels of RB, Drp1, and p-MLKL with the brown intensity of IHC images were quantified by using ImagePro-plus 6.0. **f** HE staining of sections of liver was shown. The scale bar is 50 μm. **g** The activities of AST and ALT were detected after CdCl_2_ exposure with/without metformin. Data are expressed as the mean ± standard deviation (SD). *P* < 0.05, * significantly different from Ctrl group, ^#^ significantly different from CdCl_2_ treatment.

**Supplementary Fig. S9:**

**
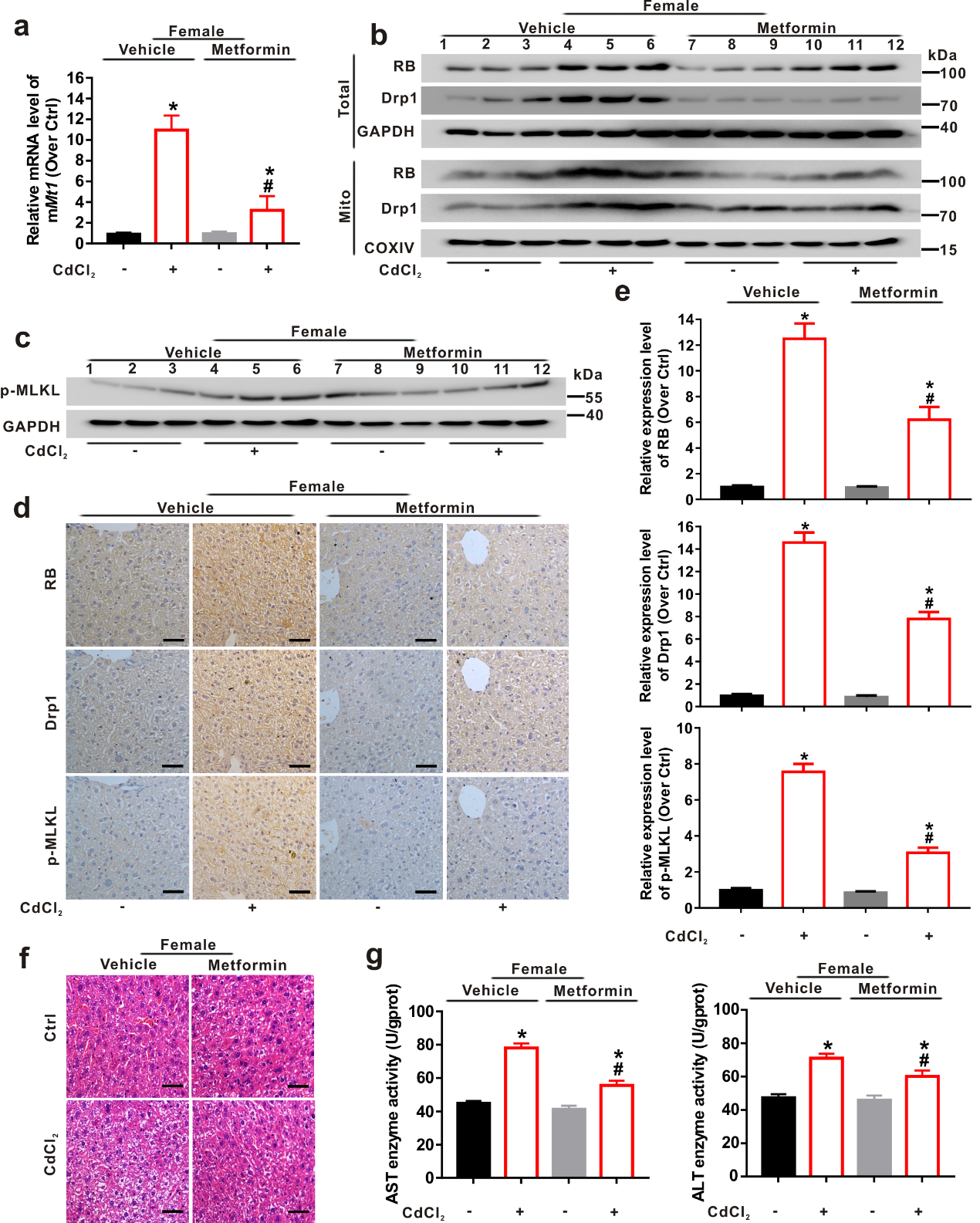
 Supplementary Fig. S9 Metformin regulates Drp1-RB axis and rescues CdCl_2_-induced hepatic necroptosis and liver injury in female mice.** The ICR female mice were intragastrically administrated with metformin (100 mg/kg bw) and intraperitoneally injected with CdCl_2_ (1 mg/kg bw) every day for one week. Vehicle was physiological saline as a solvent control. n = 3. **a** The mRNA level of m*Mt1* gene was detected by using qRT-PCR. m*Gapdh* was used as the reference gene. **b** Total lysates and mitochondrial fraction of liver tissues were prepared for SDS-PAGE with the indicated antibodies, the expression levels of RB, Drp1, and p-MLKL were evaluated by using Western blot. **c** p-MLKL protein was evaluated by using Western blot. **d-e** IHC assays with serial sections of liver were observed for tissue distribution of RB, Drp1, and p-MLKL with the indicated antibodies. **d** The representative IHC images were shown. **e** The relative expression levels of RB, Drp1, and p-MLKL with the brown intensity of IHC images were quantified by using ImagePro-plus 6.0. **f** HE staining of sections of liver was shown. The scale bar is 50 μm. **g** The activities of AST and ALT were detected after CdCl_2_ exposure with/without metformin. Data are expressed as the mean ± standard deviation (SD). *P* < 0.05, * significantly different from Ctrl group, ^#^ significantly different from CdCl_2_ treatment.

**Supplementary Fig. S10:**


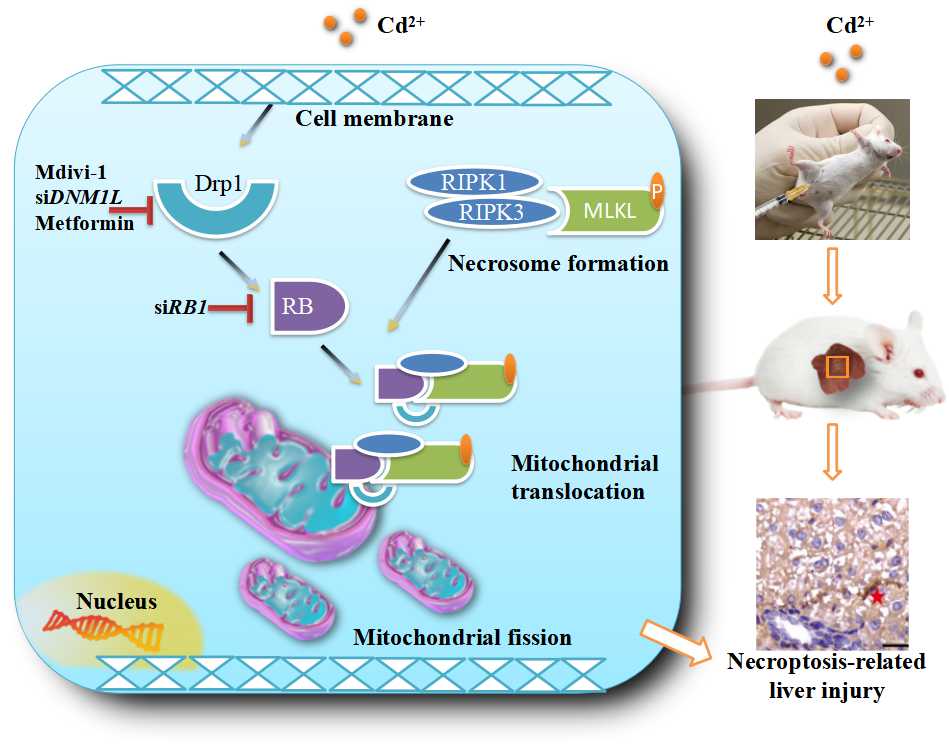


**Supplementary Fig. S10 A schematic diagram of mitochondrial Drp1 and RB interaction to mediate necroptosis induced by cadmium in hepatocytes.**

**Supplementary Fig. S11** The original full scan of each WB (as shown below, right panels) and the cropped area had been indicated (in red rectangle). Western blot bands were visualized using the Azure Biosystems or X-ray films.

**Fig 1.**


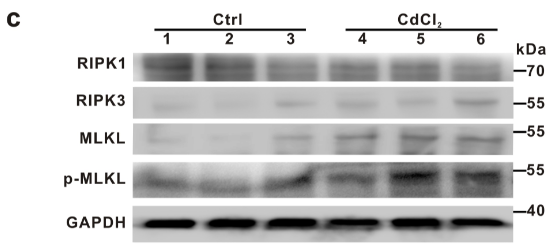

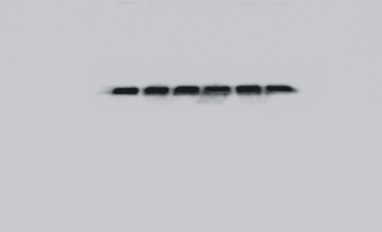

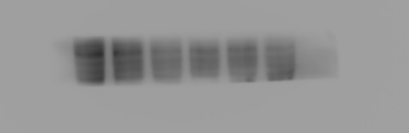

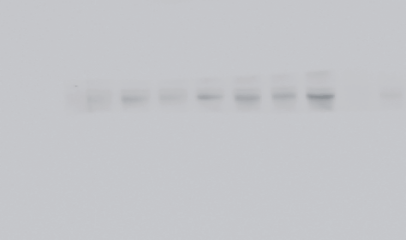

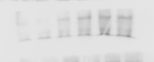

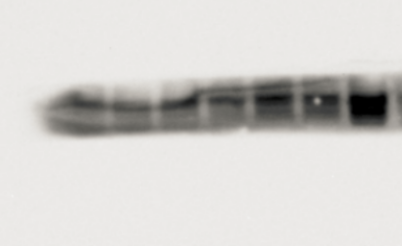


RIPK1

RIPK3

MLKL

P-MLKL

GAPDH

**Fig 2.**


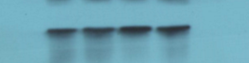

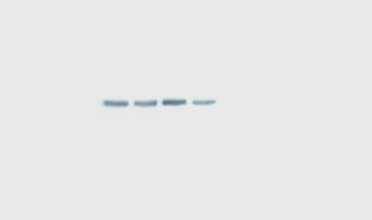

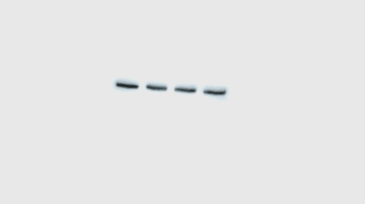

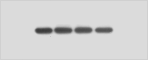

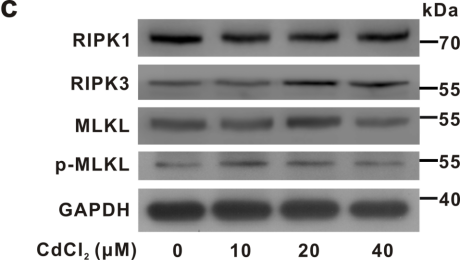

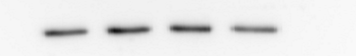


RIPK1

MLKL

RIPK3

p-MLKL

GAPDH

**Fig 3.**


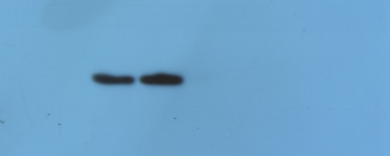

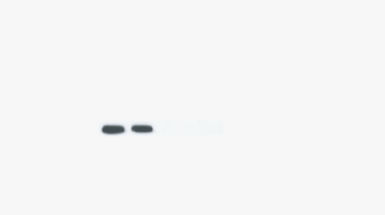

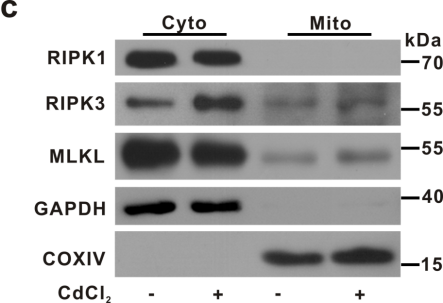

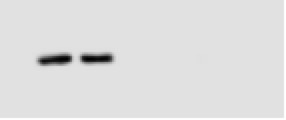

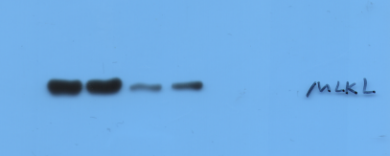

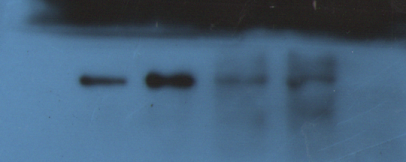


RIPK3

GAPDH

COX IV

MLKL

RIPK1

**Fig 3.**


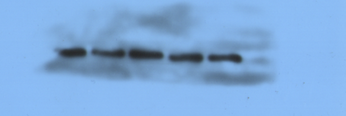

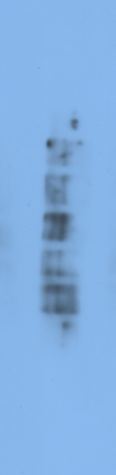

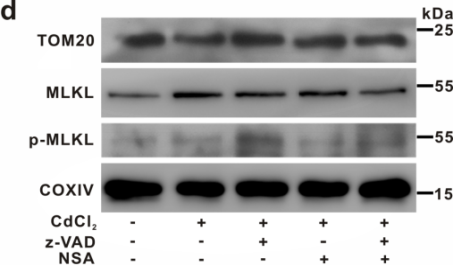

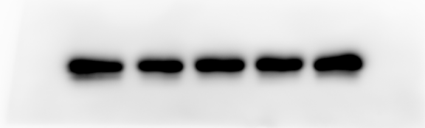

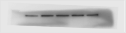


COX IV

MLKL

TTOM20

p-MLKL

**Fig 3.**

**Fig 4.**


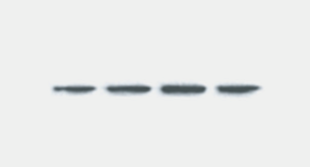

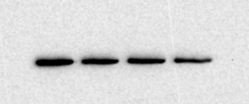

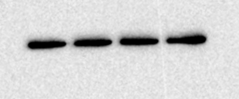

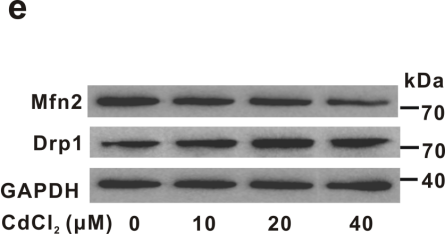


GAPDH

Drp1

Mfn2

Input

RIPK3

RIPK3


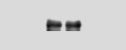

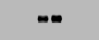

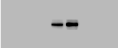

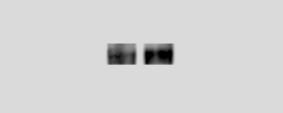

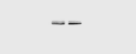

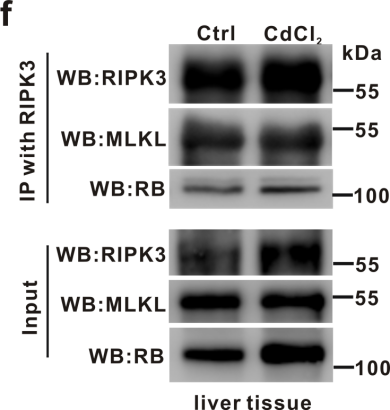

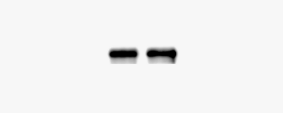


MLKL

MLKL

RB

RB

**Fig 4.**

**Fig 4.**


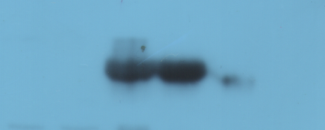

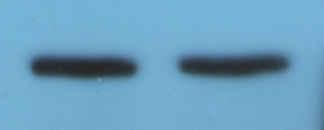

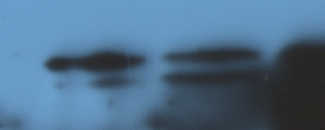

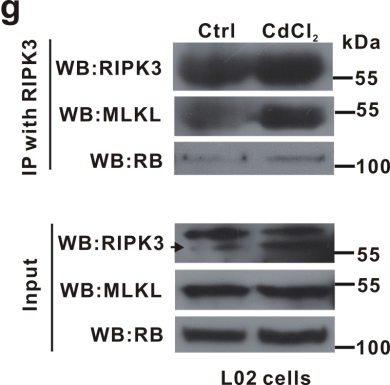

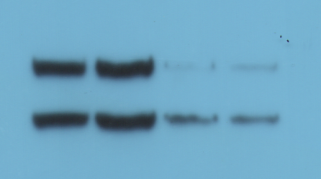

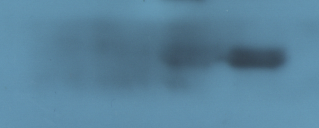


RIPK3

MLKL

RB

Input

RIPK3

MLKL

Drp1


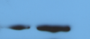

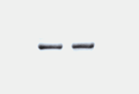

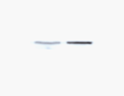

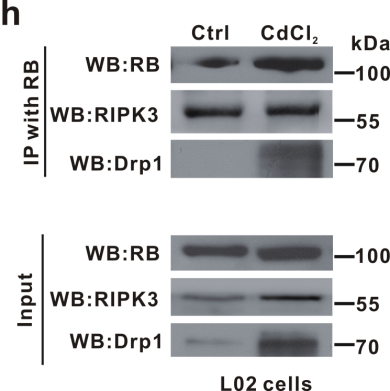

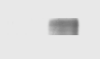

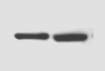

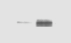


Input

RB

RIPK3

RIPK3

RB

Drp1

**Fig 5.**

**Fig 5.**

Drp1


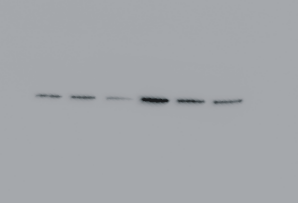

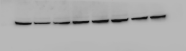

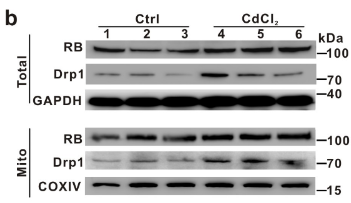

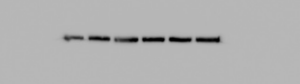


v


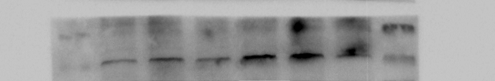


v


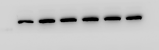


v


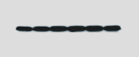


Mito

RB

CCOX IV

Total

RB

Drp1

GAPDH


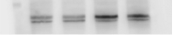

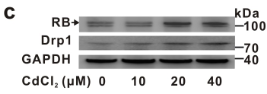

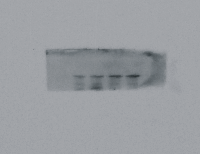

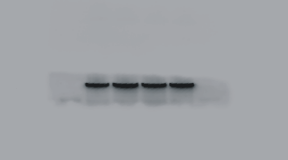


Drp1

RB

GAPDH

**Fig 5.**


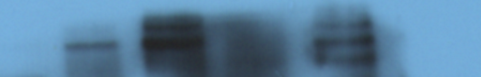

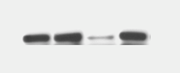

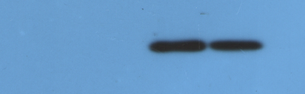

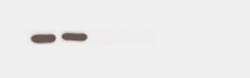

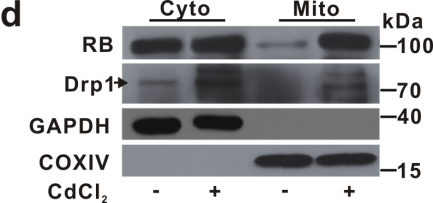


Drp1

COXIV

GAPDH

RB

**Fig 6.**


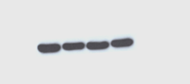

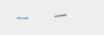

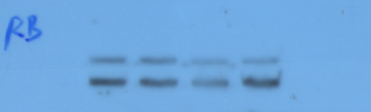

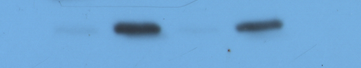

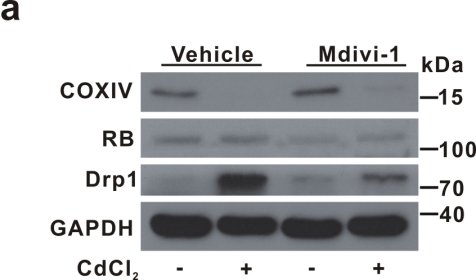


COXIV

RB

GAPDH

Drp1

**Fig 6.**

**Fig 6.**


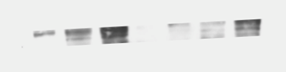

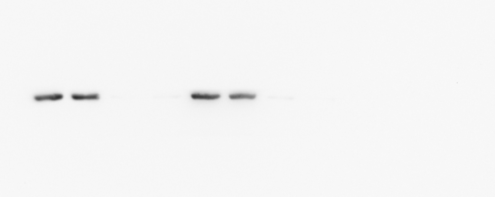

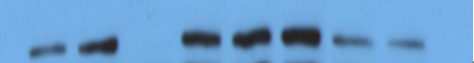

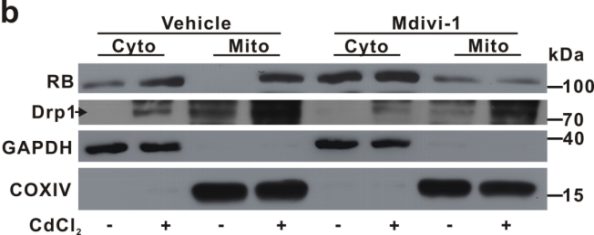

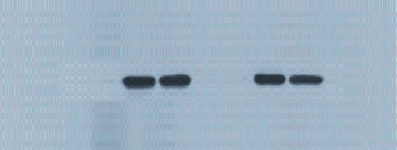


Drp1

COXIV

RB

GAPDH


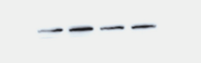

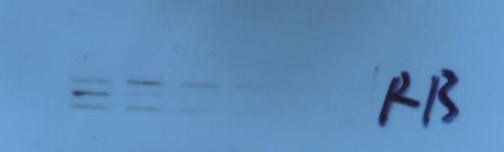

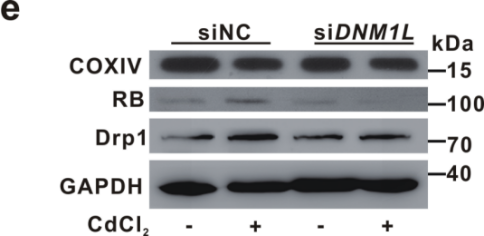

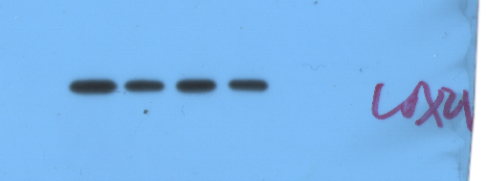

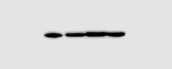


Drp1

COXIV

RB

GAPDH

**Fig 7.**


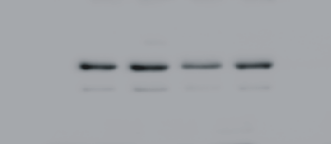

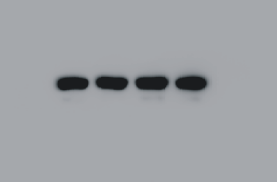

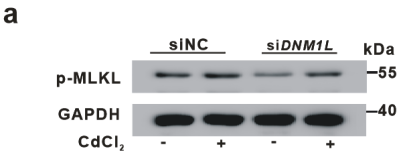


p-MLKL

GAPDH

**Fig 7.**

GAPDH


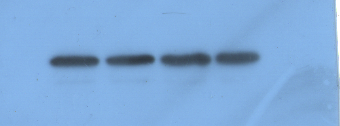

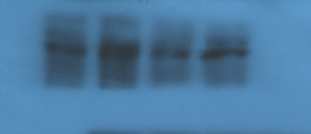

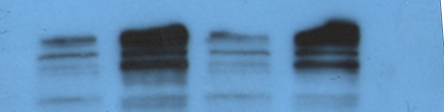

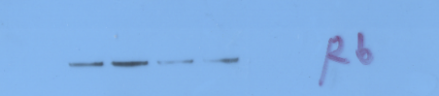

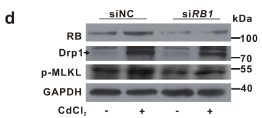


Drp1

RB

p-MLKL

GAPDH

**Fig 7.**


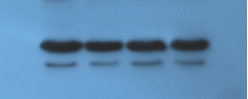

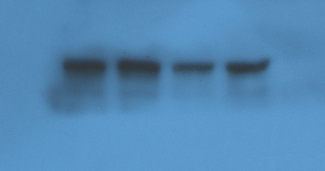

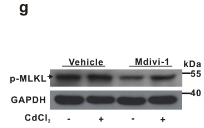


p-MLKL

**Fig 7.**


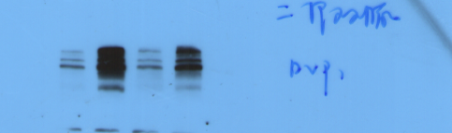


Drp1

RB

p-MLKL

GAPDH

**Fig 8.**

Drp1

RB

GAPDH

COXIV

Drp1

RB

**Fig 8.**

p-MLKL

GAPDH

**Figure S1.**

COXIV

p-MLKL

GAPDH

**Figure S1.**

p-MLKL

GAPDH

COXIV

MLKL

**Figure S2.**

p-MLKL

MLKL

RIPK1

RIPK3

GAPDH

**Figure S5.**

v

v

HHistone3

RB

GAPDH

**Figure S8.**

v

v

v

v

v

v

v

p-MLKL

GAPDH

COXIV

Drp1

RB

GAPDH

Drp1

RB

**Figure S9.**

RB

v

Drp1

v

v

GAPDH

RB

v

Drp1

v

v

COXIV

p-MLKL

GAPDH
